# Chemoenzymatic Production of Enantiocomplementary 2-Substituted 3-Hydroxycarboxylic Acids from L-α-Amino Acids

**DOI:** 10.1002/adsc.202100145

**Published:** 2021-03-22

**Authors:** Mathias Pickl, Roser Marín-Valls, Jesús Joglar, Jordi Bujons, Pere Clapés

**Affiliations:** a Department of Chemical Biology. Institute for Advanced Chemistry of Catalonia (IQAC-CSIC), Jordi Girona 18–26, 08034 Barcelona, Spain; b Department of Chemistry, University of Graz, Heinrichstrasse 28, 8010 Graz, Austria

**Keywords:** Biocatalysis, Aldol Reaction, Enzymatic Cascade, Amino Acids, Deaminase, Carboligase, 3-Hydroxycarboxylic acids

## Abstract

A two-enzyme cascade reaction plus in situ oxidative decarboxylation for the transformation of readily available canonical and non-canonical L-α-amino acids into 2-substituted 3-hydroxy-carboxylic acid derivatives is described. The biocatalytic cascade consisted of an oxidative deamination of L-α-amino acids by an L-α-amino acid deaminase from *Cosenzaea myxofaciens*, rendering 2-oxoacid intermediates, with an ensuing aldol addition reaction to formaldehyde, catalyzed by metal-dependent (*R*)- or (*S*)-selective carboligases namely 2-oxo-3-deoxy-l-rhamnonate aldolase (YfaU) and ketopantoate hydroxymethyltransferase (KPHMT), respectively, furnishing 3-substituted 4-hydroxy-2-oxoacids. The overall substrate conversion was optimized by balancing biocatalyst loading and amino acid and formaldehyde concentrations, yielding 36–98% aldol adduct formation and 91– 98% ee for each enantiomer. Subsequent in situ follow-up chemistry via hydrogen peroxide-driven oxidative decarboxylation afforded the corresponding 2-substituted 3-hydroxycarboxylic acid derivatives.

## Introduction

**Scheme 1:**
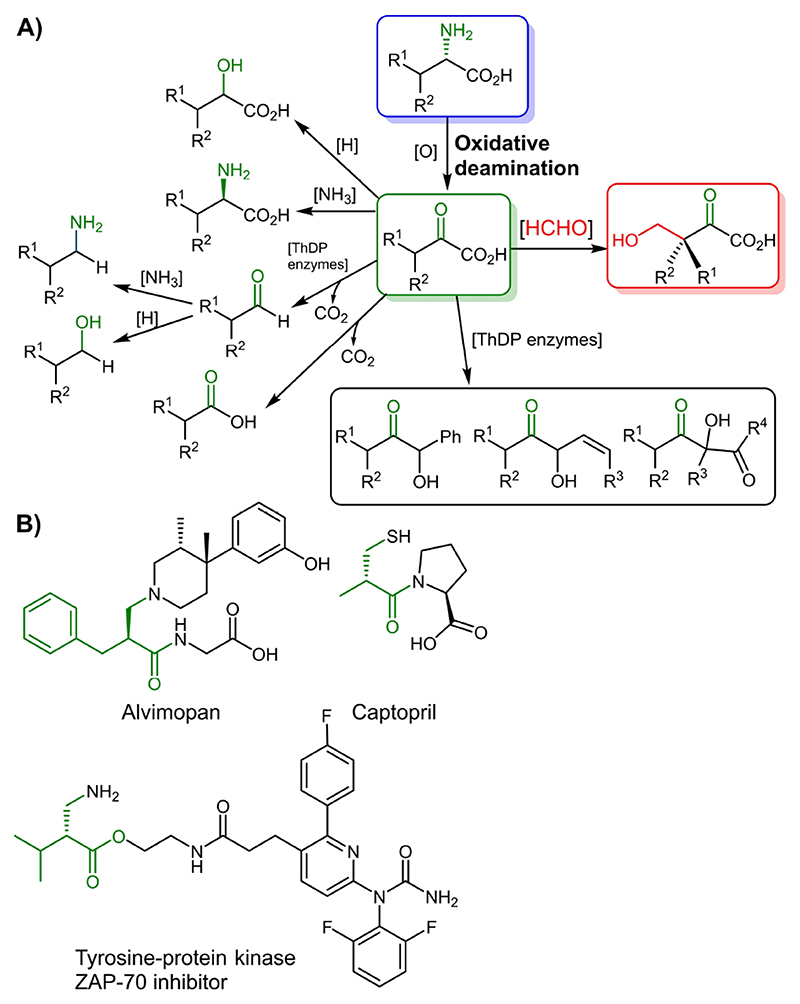
A) Oxidative deamination of L-α-amino acids to 2-oxoacids, key intermediates for a variety of functional groups. B) Examples of bioactive compounds bearing moieties that can be built up by 3-hydroxymethylcarboxylic acid derivatives.

The high selectivity of biocatalysts in functionalization reactions facilitates the transformation of bio-based molecules into intermediates that lead to the production of high value chemicals.^[1]^ For example, via oxidative deamination of L-α-amino acids, 2-oxoacids are accessible and these can act as suitable intermediates for a broad range of potential products^[2]^ and enable further remarkable transformations (Scheme 1A).^[3]^ The L-α-amino acids are produced in large scale fermentation, a process which is subjected to continuous improvement of their cost-effective manufacturing.^[4]^ Deamination reactions can be performed by a plethora of established biocatalysts, among them α-transaminases (ATAs, EC: 2.6.1.x),^[2h,5]^ amino acid dehydrogenases, (AADHs, EC: 1.4.1.x),^[6]^

l-amino acid oxidases (l-AAOs, EC: 1.4.3.2)^[7]^ or amino acid deaminases (AADs, EC: 1.4.99.B3).^[8]^ Interestingly, the essential irreversibility provided by oxidase-like enzymes (EC: 1.4.x.x) is highly beneficial, as equilibrium sensitive downstream reactions are facilitated.^[2h]^

2-Substituted 3-hydroxycarboxylic acid or ester derivatives are among the most relevant classes of compounds that may be achieved from 2-oxoacid intermediates.^[9]^ 2-Substituted 3-hydroxycarboxylic acids or their ester derivatives also serve as versatile building blocks in a wide range of active ingredients in pharmaceutical applications such as in Alvimopan, an FDA approved drug used for the treatment of post-operative ileus, Captopril, an angiotensin-converting enzyme inhibitor, or a tyrosine—protein kinase ZAP-70 inhibitor, (Scheme 1B).^[10]^

In a previous work we developed an effective chemoenzymatic method for the synthesis of enantio-merically pure 2-substituted 3-hydroxycarboxylic esters.^[9b]^ The key step was an enzymatic stereo-selective aldol addition of chemically synthetized 2-oxoacids to formaldehyde catalyzed by two enantio-complementary Type II 2-oxoacid aldolases, 2-keto-3-deoxy-l-rhamnonate aldolase (YfaU, EC 4.1.2.53) fused with maltose binding protein from *E. coli* (MBP) (MBP-YfaU), and 3-methyl-2-oxobutanoate hydroxymethyltransferase (KPHMT, EC 2.1.2.11) and variants thereof. The homochiral 3-substituted 4-hydroxy-2-oxoacids produced were transformed into 2-substituted 3-hydroxycarboxylic ester derivatives by oxidative decarboxylation in the presence of hydrogen peroxide and chemical esterification.

Our ongoing work on the synthesis of 2-substituted 3-hydroxycarboxylic acids and derivatives has prompted us to develop an enzymatic cascade strategy for their preparation comprising: *i*) an enzymatic deamination reaction of L-α-amino acids rendering 2-oxoacids; *ii*) an ensuing aldol addition of 2-oxoacid to formaldehyde catalyzed by MBP-YfaU and KPHMT, furnishing both enantiomers of 3-substituted 4-hydroxy-2-oxoacids, and *iii*) in situ transformation into 2-substituted 3-hydroxycarboxylic acids by oxidative decarboxylation. This strategy represents a complementary approach for the synthesis of 3-hydroxycar-boxylic acids using widely available and diverse canonical and non-canonical L-α-amino acids as starting materials.

## Results and Discussion

**Scheme 2:**
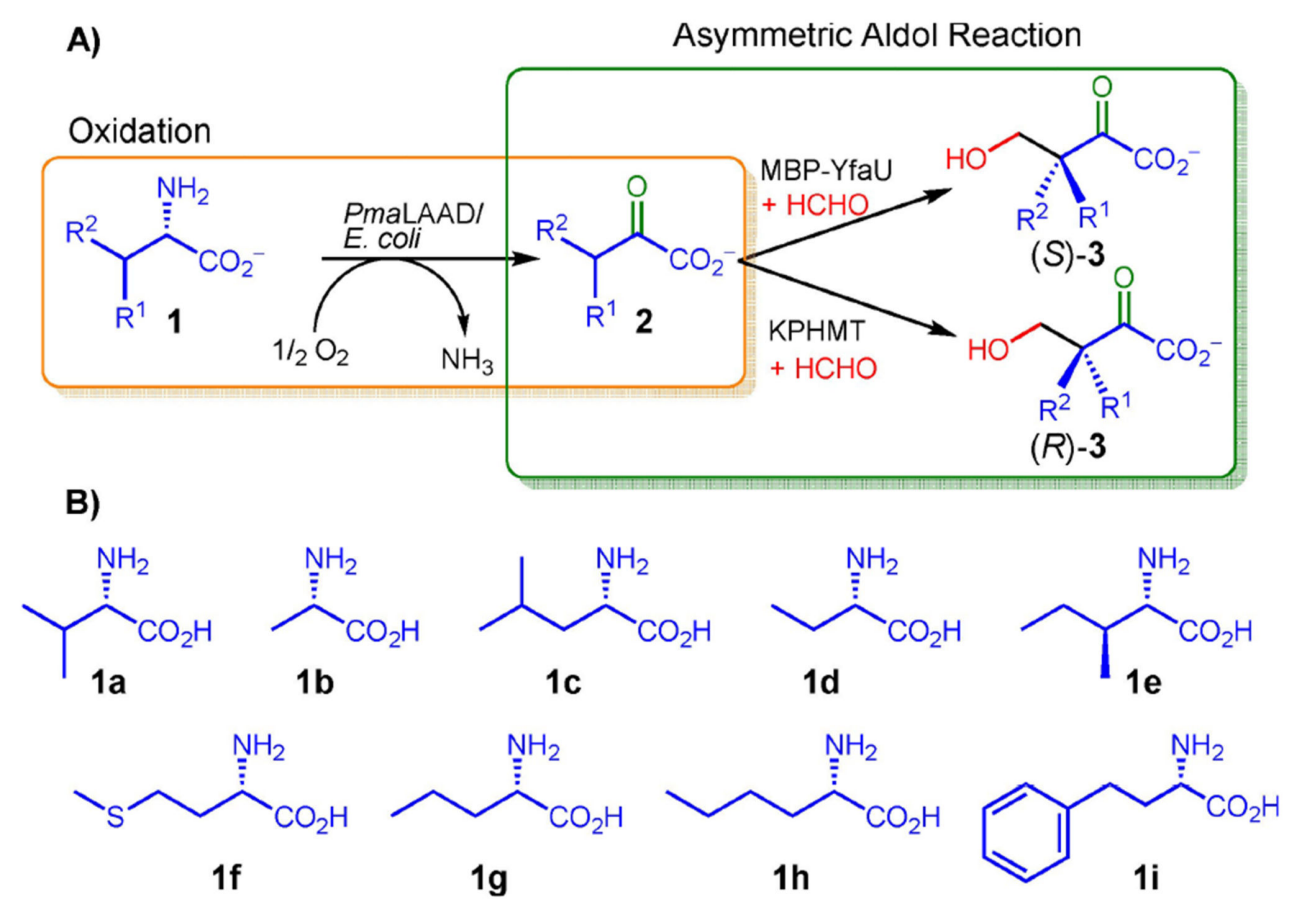
A) Two-enzyme cascade system for the synthesis of enantiomerically enriched (3*S*)- or (3*R*)-substituted 4-hydroxy-2-oxoacids (3 a–i) starting from L-α-amino acids 1 a–i, via 2-oxoacid intermediates 2 a–i. B) Substrate panel of canonical and non-canonical L-α-amino acids 1.

A two-enzyme cascade reaction setup was envisioned for the preparation of enantiocomplementary 3-sub-stituted 4-hydroxy-2-oxoacid precursors **3** from L-α-amino acids **1** (Scheme 2).

For the oxidative deamination of **1**, the membrane associated flavoenzyme l-amino acid deaminase from *Proteus myxofaciens*, later classified as *Cosenzaea myxofaciens* ^[11]^ (*Pma*LAAD, EC 1.4.99.B3), was selected as the catalyst.^[8a,b,12]^ This enzyme yields the corresponding 2-oxoacid and ammonia from L-α-amino acids and O_2_, without the cumbersome formation of hydrogen peroxide that normally occurs in oxidase driven reactions.^[12b]^ This is the case because the electrons of the reduced cofactor FADH_2_ of *Pma*LAAD, are transferred first to cytochrome *b*-like proteins and then through the respiratory chain to O_2_ yielding H_2_O.^[8a,b]^ A membrane associated respiratory chain is thus required for the catalysis, and thus *Pma*LAAD was employed as freeze-dried *E. coli* cells harbouring the overexpressed enzyme. This configuration revealed good results on a panel of L-α-amino acids (see SI, Figure S1) making this enzyme adequate in the envisioned cascade reaction setup. Furthermore, due to the use of O_2_ as oxidant, the catalyst is redox self-sufficient, thus, it is a perfect partner in combination with non-redox carboligation reactions.^[8a]^

For the stereoselective aldol addition, MBP-YfaU, KPHMT and variants thereof, offer a wide acceptance toward diverse 2-oxoacids.^[9b,13]^ Thus, wild-type MBP-YfaU and variant W23V, supplied with Ni^2_^ as metal cofactor,^[14]^ were selected for the (*S*)-selective aldol reaction, while wild-type KPHMT and variants I202A and I212A supplied with Co^2+^ were the biocatalysts of choice for the preparation of the (*R*)-aldol products.^[9b]^

**Figure 1:**
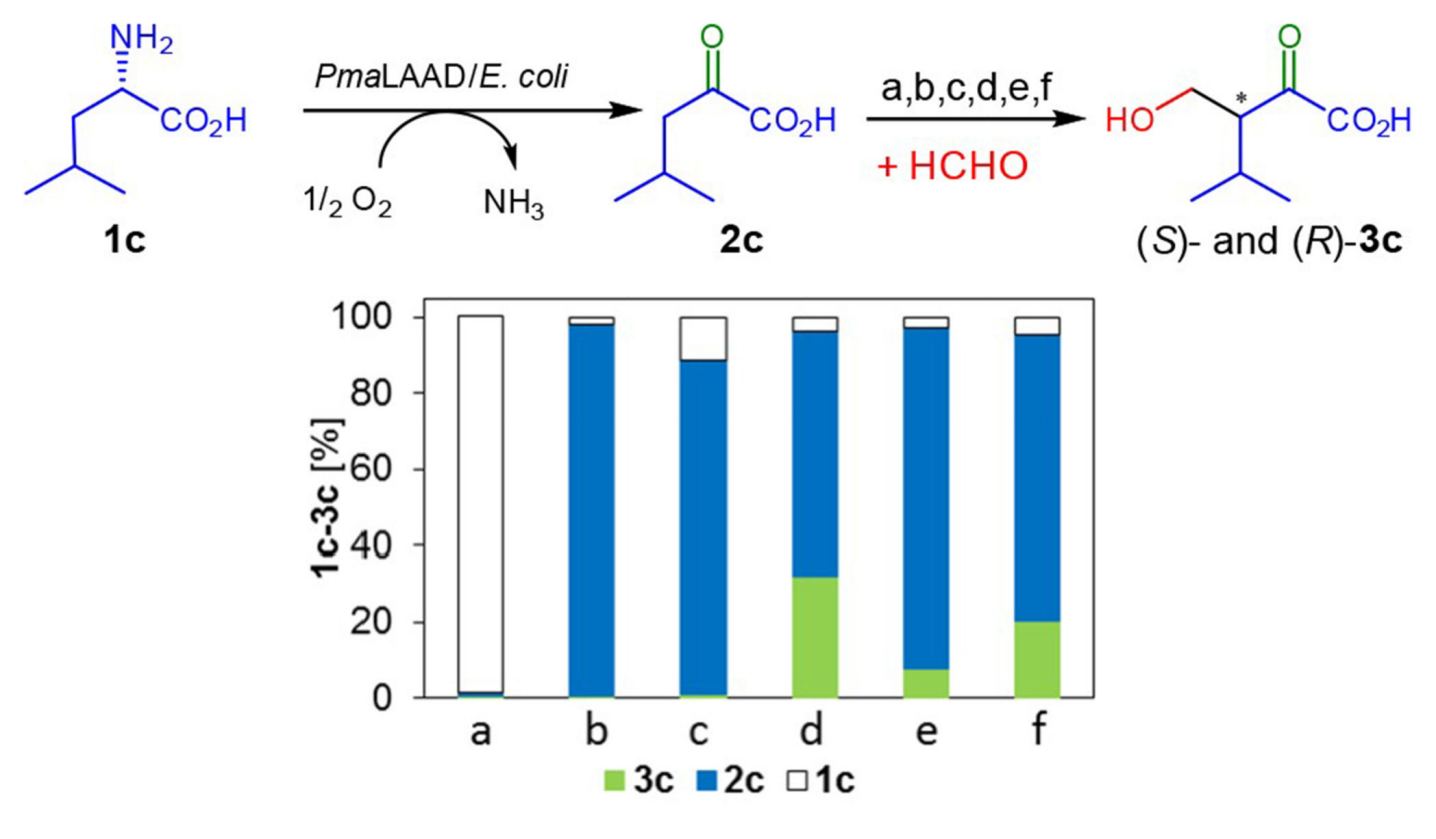
Comparison of the deamination-aldol cascade under different catalyst set up. X-axis: a: non-transformed freeze-dried *E. coli* cells in the presence of aldolase, b: no aldolase, c: wild-type MBP-YfaU, d: MPB–YfaU-W23V, e: wild-type KPHMT, f: KPHMT-I202A. Conditions: 50 mM **1 c**, 50 mM formaldehyde, 20 mg freeze-dried cell preparation, 1 mg carboligase (MBP-YfaU: 0.03 mol%, KPHMT 0.04 mol%), 1 mL MilliQ water, rt, horizontal shaking. Formation and remaining reaction components **1 c**, **2 c** and (*S*)-and (*R*)-**3 c** were analyzed after 24 h of reaction.

In an initial control experiment to verify *Pma-*LAAD activity, non-transformed freeze-dried *E. coli* cells were tested on the model substrate **1 c** (Figure 1) in the cascade reaction setup. Only negligible trace activity was observed from the *E. coli* background. Moreover, no aldol addition was observed in the absence of the carboligase, and consequently only 2-oxoacid **2 c** was accumulated. Initial tests with the carboligases were promising as the conversion of **1 c** in a cascade fashion furnished 32% of (*S*)-**3 c** and 20% of (*R*)-**3 c** after 24 h, employing MBP-YfaU-W23V and KPHMT-I202A catalysts, respectively, with equimolar (50 mM) concentration of **1 c** and formaldehyde.

To improve the formation of the target aldol products, several parameters were screened for establishing optimal conditions. Catalyst loading turned out to be not crucial since aldol formation was about the same (20–30%) for enzyme loadings between 1 and 8 mg mL ^1^ (see SI, Figure S3). Performing the reaction in a one-pot two-steps process also had negligible influence on the overall outcome (see SI, Figure S4). Moreover, a prominent negative factor was found to be the formation of ammonia in the first cascade step, which reduced the activity of the aldolase (see SI, Figure S6). The cascade system turned out to tolerate several organic co-solvents, but no significant improvement was achieved (see SI, Figure S5). Most importantly, the concentration of formaldehyde showed to have the most crucial effect on aldol formation. Several examples are described showing that the supply of the aldol acceptor in excess is beneficial for the product formation of reactions mediated by aldolases related to MBP-YfaU.^[15]^ Consequently, an excess of formaldehyde at various concentrations of substrate **1 c** was explored (Table 1) with variant MBP-YfaU-W23V in the cascade setup. It turned out that with a **1 c** loading of 10 mM and 15 equivalents (150 mM) of formaldehyde, the aldol product formation was boosted to 78% (Table 1, Entry 6). Therefore, these were the conditions chosen for further investigations. The highest productivity (24 mM) was reached with a substrate load of 75 mM and 150 mM of formaldehyde (Table 1, Entry 9) despite it only represented a 32% conversion. Conversions were increased at higher formaldehyde:**1 c** ratios, *i.e.* from 1:1 (Table 1, entry 2) to 15:1 (Table 1, entry 6). However, aldol formation dropped at the highest formaldehyde concentrations assayed (Table 1, entries 10 and 11), which may be caused by inactivation of *Pma*LAAD.

**Figure 2:**
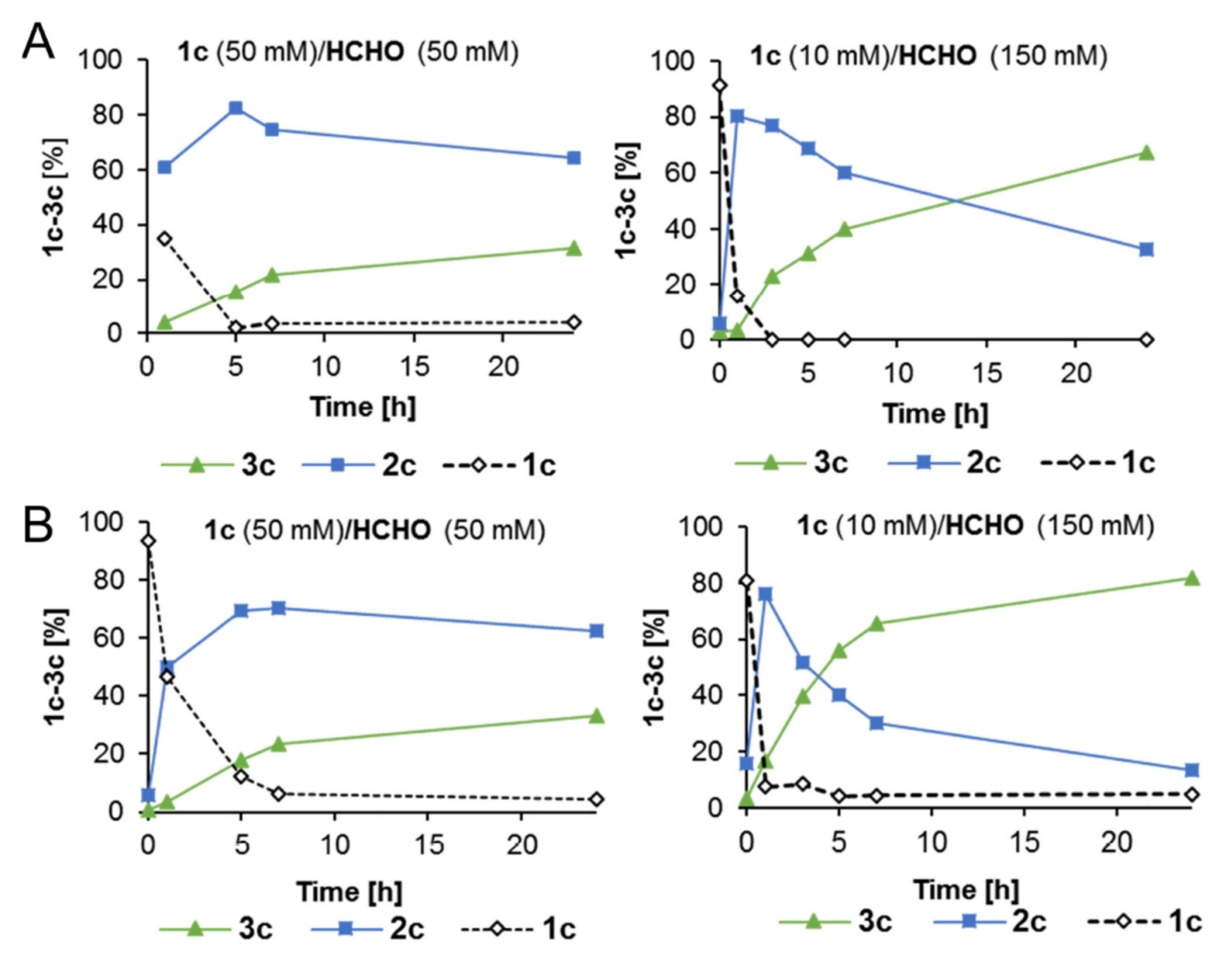
Substrate 1 c conversion to aldol product (*S*)- and (*R*)-3 c employing *Pma*LAAD and carboligases. A) *S*-selective Type II aldolase variant MBP-YfaU-W23V, B) *R*-selective KPHMT-I202A variant. Conditions: lyophilized whole cells *Pma*LAAD (1.1U), MBP-YfaU-W23V (2 mg) or KPHMT-I202A (1 mg), NiCl_2_ or CoCl_2_ (0.6 mM), respectively, MilliQ water (1 mL), rt, horizontal shaking, 24 h. Left: 50 mM substrate, 50 mM formaldehyde; Right: 10 mM substrate, 150 mM formaldehyde.

Next, a comparative study of aldol product formation *versus* reaction time was performed using (*i*) initial equimolar conditions of formaldehyde and **1 c**, and (*ii*) the optimized formaldehyde and **1 c** concentrations, *i.e.* 150 mM and 10 mM, respectively. The results revealed that under the optimized conditions, product formation significantly increased within the reaction time of 24 h (Figure 2). Full l-amino acid conversion was achieved after 3 h, regardless of the subsequent carboligase used, *i.e.* MBP-YfaU-W23V or KPHMT-I202A. Overall, both carboligases showed negligible effect on the *Pma*LAAD performance and rely on formaldehyde supply for aldol product formation. Indeed, formaldehyde concentration appears to be crucial to shift the equilibrium towards the aldol adduct in the second step, and the potential negative effect of ammonia released during the deamination step was negligible.

Hence, the substrate panel was tested under the established optimized conditions with wild-type MBP-YfaU, MBP-YfaU-W23V, wild-type KPHMT, KPHMT-I202A and KPHMT-I212A catalysts (Table 2), which successfully probed activity on several intermediates **2**.^[9b]^

The substrate scope of the cascade system turned out to be strictly limited to non-functionalized α-amino acids (see SI, Figure S8), with a preference for medium chain lengths as **1 d**, and **1 g**–**h**, however, tolerating an internal heteroatom (**1 f**) or the rather bulky benzyl substituent (**1 i**). Ramification in position 3 (**1 a** and **1 e** rendering **2 a** and **2 e**, respectively) was tolerated by all KPHMT catalysts (Table 2, entries 1–3 and 16–18), whereas with MBP-YfaU catalysts only negligible aldol was formed (Table 2, entries 4–5 and 19–20). The shortest amino acid **1 b** was not accepted by *Pma*LAAD in accordance with the literature,^[8a]^ even though the 2-oxoacid **2 b** is known to be a well-accepted substrate by both MBP-YfaU and KPHMT.^[9b]^ The highest overall conversions were achieved employing unbranched aliphatic α-amino acids **1 d**, **1 g** and **1 h**, reaching almost complete conversion to the aldol product in some cases (Table 2, entries 14, 15, 26, 28, 30, 33 and 35).

Although l-phenylalanine derivatives are known to be readily deaminated to their corresponding 2-oxoacids by *Pma*LAAD,^[16]^ the non-enzymatic reaction background of the easily enolizable phenylpyruvic acid derivatives yielded only the racemic product (data not shown). However, using the homolog homophenylalanine (**1 i**), in which the phenyl group is distant from the enolizable carbonyl functionality present in the corresponding intermediate **2 i**, the background reactivity was abolished and both enantiomers of the corresponding product **3 i** were accessible with high conversion (Table 2, entries 38 and 40).

**Scheme 3:**
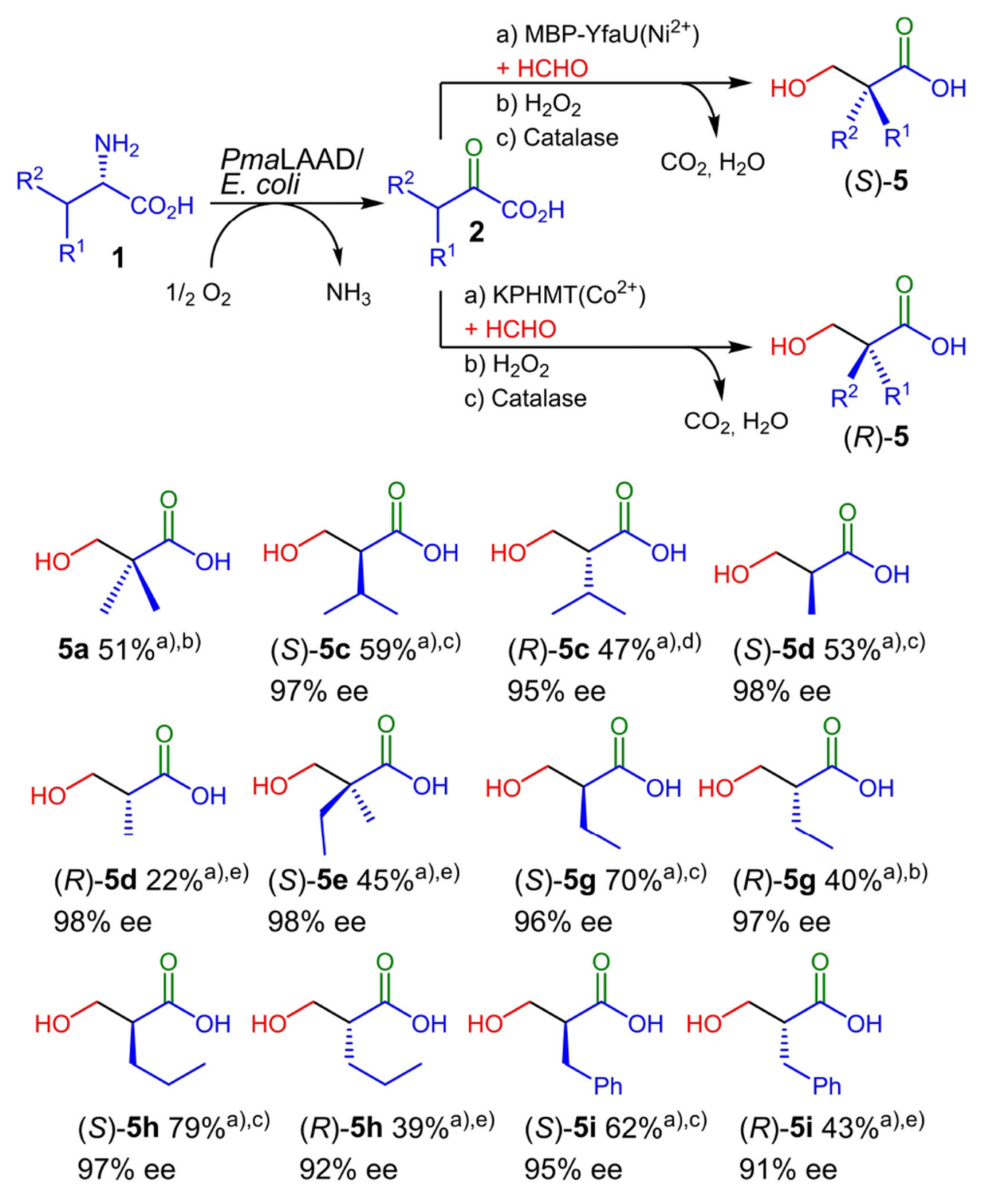
Yields and enantiomeric excesses of 5 a, 5 c–e and 5 g–i in the preparative scale experiments. ^a^Isolated yield. ^b^Wild-type KPHMT. ^c^MBP-YfaU-W23V. ^d^KPHMT-I202A. ^e^KPHMT-I212A.

With the best variants and conditions found to maximize substrate conversion, we checked the applicability of the enzymatic cascade at preparative scale. For the sake of catalyst stabilization, the reaction medium was switched to a borate buffer (50 mM, pH 7.5), which has negligible background activity (*i. e.* non-enzymatic reaction, data not shown) contrary to other buffer systems (*i. e.* phosphate buffer).^[9b]^ After performing the cascade in a 50 mL batch scale (0.5 mmol respect to the limiting l-amino acid), the aldol products **3** were treated in situ with H_2_O_2_ yielding 2-substituted 3-hydroxycarboxylic acids **5** isolated by simple extraction without any further purification in 22–79% yields (Scheme 3). Compound **5 f** could not be obtained because the oxidative decarboxylation was not compatible with the thioether moiety of the aldol product **3 f**. Formation of the corresponding sulfoxide derivatives plus other unidentified degradation compounds were mostly the result of H_2_O_2_ treatment, similarly to what occurred with methionine.^[17]^

**Scheme 4:**
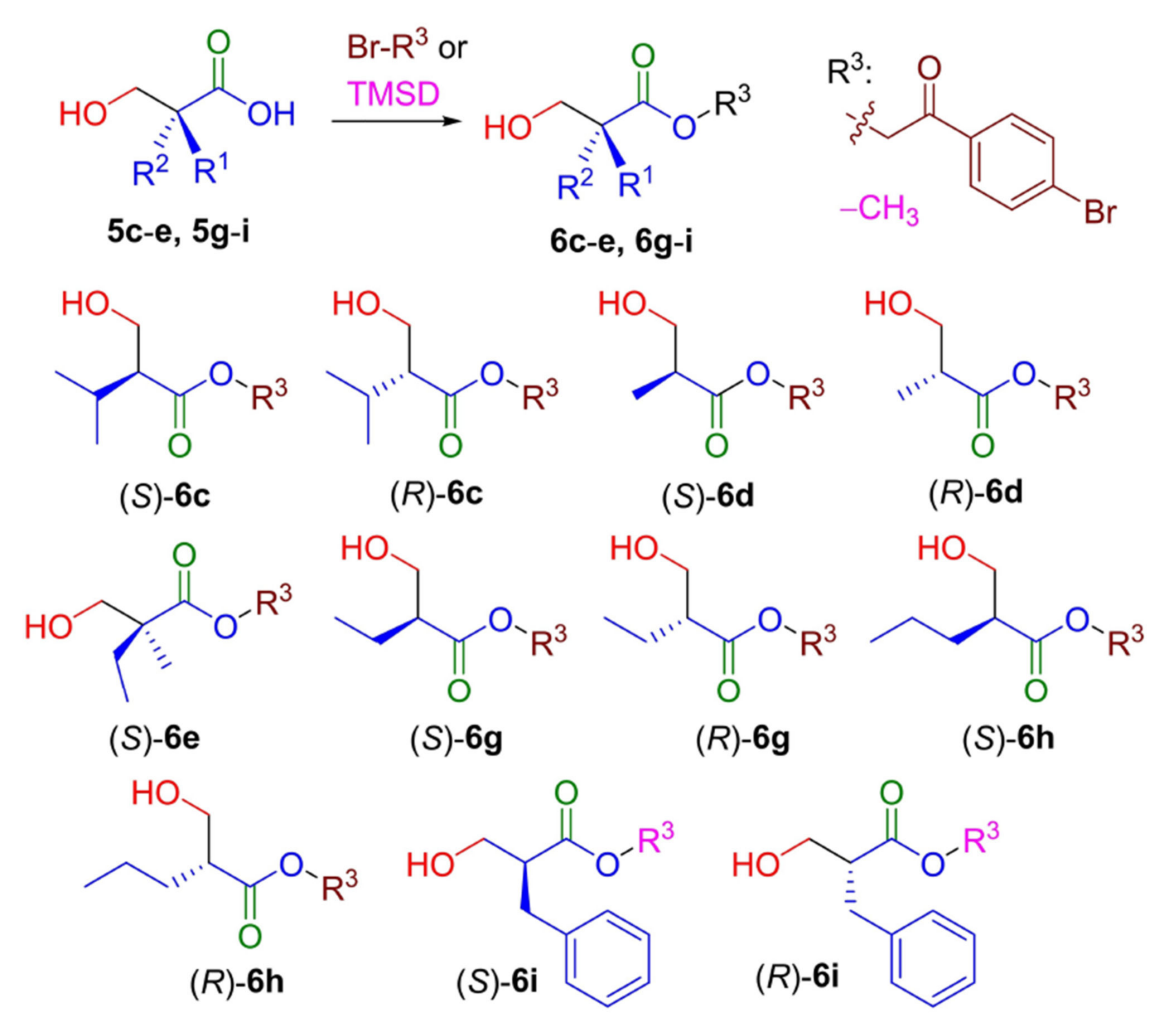
Esterification of products 5 with 2,4’-dibromoaceto-phenone or trimethylsilyldiazomethane yielding compounds 6 for HPLC analyses on chiral stationary phase.

The ees were determined by HPLC analyses on chiral stationary phase after pre-column esterification with 2,4’-dibromoacetophenone or tri methylsilyldiazomethane for compounds **5 i** (Scheme 4). The absolute configuration was verified by comparing the optical rotation with literature data (see SI, Table S3), as well as by the elution order obtained in the chiral stationary phase-HPLC chromatograms of derivatives **6**.^[9b]^ Both enantiomers of products **6 c**–**d** and **6 g**–**i** were achieved with 91 to 98% ee. For **6 e** only the *S*-enantiomer was obtained, as found in a previous publication.^[13b]^ As we stated before, the oxidative decarboxylation was not compatible with the thioether moiety of the aldol product **3 f**. Thus, the derivatization protocol could not be performed with **3 f**.

## Conclusions

In summary, a biocatalytic cascade is described in which both enantiomers of the highly versatile 4-hydroxy-2-oxoacids are produced out of canonical and non-canonical L-α-amino acids. The key oxidative deamination step performed by *Pma*LAAD only requires molecular oxygen, while the aldol addition to formaldehyde in the second step enables the access to both enantiomers of 4-hydroxy-2-oxoacid derivatives using an appropriately selected carboligase. The key feature to yield the aldol product with high overall conversion was a large excess of the aldol acceptor that shifts the reaction equilibrium of the aldol step to the corresponding adduct. The results successfully demonstrated that the designed two enzyme cascade can serve as a tool to derive building blocks for pharmaceutical applications directly from abundant canonical and non-canonical L-α-amino acids.

The strategy afforded aldol products that are suitable precursors for a variety of chemo-enzymatic transformations. The selected route towards 2-substituted 3-hydroxycarboxylic acid derivatives produces relevant building blocks for pharmaceutical active compounds. This protocol offers a benign alternative to established methods, providing both enantiomers in good yields.

## Experimental Section

### Materials

All amino acids except l-leucine and l-phenylalanine were purchased from TCI (Zwijndrecht, Belgium), l-leucine from Merck (Darmstadt, Germany), hydrogen peroxide solution from Scharlab (Sentmenat, Spain), and l-phenylalanine, 2-oxoacid standards, and formaldehyde solution from Sigma Aldrich (Steindorf, Germany), and used without further purification. High-density IDA-Agarose 6BCL nickel charged was from GE Healthcare Life Science. Water for analytical HPLC and for the preparation of buffers and other assay solutions was obtained from an Arium pro ultrapure water purification system (Sartorius-Stedim Biotech). All other solvents used were of analytical grade.

### General Methods

Thin layer chromatography was performed using precoated silica gel plates with or without fluorescent indicator UV_254_ (Macherey-Nagel GmbH & Co. KG, Kieselgel 60). Column chromatography was performed in a glass column (AFORA, 5880/2, 47×4.5) packed with silica gel (100 g, 35–70 μm, 200– 500 mesh, Merck). Stains were detected on TLC plates using UV254 fluorescence or developed with ceric ammonium molybdate (CAM) stain (Ce(SO_4_)_2_ (10 gL^-1^) and (NH_4_)_6_Mo_7_O_24_·4H_2_O (50 gL^-1^) in H_2_SO_4_ 2 M).

#### Specific rotation

Specific rotation values were measured with a Perkin Elmer Model 341 (Überlingen, Germany).

#### NMR analysis

Routine, ^1^H (400 MHz) and ^13^C (101 MHz) NMR spectra of compounds were recorded with a Varian Mercury-400 spectrometer. Full characterization of the described compounds was performed using typical gradient-enhanced 2D experiments: COSY, HSQC, NOESY and HMBC recorded under routine conditions.

### Analytical Methods

#### HPLC reaction monitoring

HPLC analysis were performed on an RP-HPLC XBridge^®^ C18, 5 μm, 4.6 × 250 mm column (Waters). The used solvent system was: solvent (A): 0.1% (v/v) trifluoroacetic acid (TFA) in H_2_O and solvent (B): 0.095% (v/v) TFA in CH_3_CN/H_2_O 4:1, flow rate 1 mLmin^-1^, detection at 215 nm and column temperature at 30 °C. The amount of product and substrates was quantified from the peak areas using an external standard methodology and calibration curves.

#### Derivatization method for 2-oxo acids **2** and aldol products **3**

An aliquot of the reaction mixture (10 μL) was mixed with a solution of *O*-benzylhydroxylamine hydrochloride (50 μL of a 130 mM stock solution in pyridine/methanol/water 33:15:2). After incubation at 25 °C for 5 min, samples were diluted in methanol (500 μL) and after centrifugation (20,000 × g, 5 min) analyzed by HPLC. Elution conditions: gradient elution from 10 to 100% B over 30 min.

#### Derivatization of amino acids for reaction monitoring

After a dilution of the reaction mixture with water (1:1), an aliquot (10 μL) was mixed with a solution of *N*-(benzyloxycarbonyloxy)succinimide (CbzOSu) (50 μL of a 150 mM stock solution in acetonitrile). After incubation at 60 °C for 60 min, samples were diluted in methanol (440 μL) and after centrifugation (20,000×g, 5 min) analyzed by HPLC. Elution conditions: gradient elution from 10 to 100% B over 30 min.

#### Chiral HPLC analysis

Enantiomeric excesses (ee) were determined using HPLC analysis on chiral stationary phase in 46 × 250 mm columns, 5 μm particle size and 254 nm or 209 nm UV detection. Column type, specific elution conditions, and flow rates are described for each compound.

#### Follow up chemistry of 2-oxoacids for chiral HPLC analysis

First, the cascade deamination-aldolase reaction was carried out for each starting L-α-amino acid (see below). The reaction mixture (1 mL) was centrifuged (20,000×g, 5 min) and after discarding the pellet, hydrogen peroxide (30 μL of an 8.8 M commercial solution; 60 μL when MBP-YfaU catalysts was used and 5 μL when KPHMT catalysts were used) was added to the reaction mixture and shaken (100 rpm) in an open vessel. After the reaction was completed, catalase from bovine liver (1.25 mg, 3500 U) dissolved in sodium phosphate buffer (25 μL, 10 mM, pH 7) was added. The reaction mixture was diluted with methanol (500 μL), centrifuged, and the solvent was removed under airflow. A solution of 2,4’-dibromoaceto-phenone (14 mg) in DMF (250 μL) was added to the residue and it was shaken for 1 h (1000 rpm). Then, EtOAc (500 μL) was added to the reaction mixture and washed with H_2_O (3 × 250 μL). The organic phase was dried over MgSO_4_ and the solvent evaporated under vacuum. The residue was dissolved in hexane/*i*PrOH (75:25) and analyzed by chiral HPLC. Column type, specific elution conditions, and flow rates are described below for each compound. Derivatized compounds **6** were identified by comparing them with authentic samples prepared as described in a previous work, as well as the corresponding racemic mixtures.^[9b]^ *Derivatization of compound **5 i** for chiral HPLC analysis*: A sample (1 mg) of **5 i**, obtained using the procedure described above, was diluted in a mixture of MeOH/EtOAc (1:3, 200 μL), and then trimethylsilyldiazomethane (30 μL of a 0.6 M solution in hexane) was added. The conversion of **5 i** was controlled after 5 min incubation time. The solvent was evaporated under vacuum and the residue dissolved in hexane/^*i*^PrOH (75:25) and analyzed by chiral HPLC. Column type, specific elution conditions, and flow rates are described below. The racemic sample was prepared by spiking a sample of the (*S*)-enantiomer with the equivalent amount of the (*R*)-enantiomer. The absolute configuration of the (*R*)-enantiomer was verified by optical rotation [α]^20^_D_= + 12.7 (*c* 3.0, CHCl_3_); lit.:^[10j]^ [α]^20^_D_= + 12.5 (*c* 2.0,CHCl3)(*R*).

### Enzyme Production and Activity Tests

#### Production of l*-Amino Acid Deaminase from P. myxofaciens.*

^[12a]^ *E. coli* BL21(DE3) clones containing the *Pma*LAAD plasmid (pET21a expression plasmid, Merck, Vienna) were grown in a LB medium which was prepared by sterilizing a solution (1 L) of the following components in 5 unbaffled 2 L-flasks: Trypton (10 gL^-1^), NaCl (10 gL^-1^) and yeast extract (5 gL^-1^). A preculture was prepared by inoculating 100 mL of LB-medium containing ampicillin (100mgL^-1^). The preculture was shaken overnight at 150 rpm and 37 °C. Afterward the 2 L-flasks containing 1 L LB media with ampicillin (100mgL^-1^) were inoculated with the preculture giving an initial OD_600_ =0.05. Then, the cultures were shaken at 120 rpm and 37 °C until OD_600_=0.5–0.7 was obtained. The protein expression was induced with IPTG (0.5 mM, final concentration) and the cultures were shaken for 24 h at 25 °C and 150 rpm. Finally, the cells were harvested by centrifugation (2,500×g, 45 min), washed with potassium phosphate buffer (10 mM, pH 7), shock frozen in dry ice, and lyophilized. The lyophilized cells were stored at — 20 °C and used without further treatment for the biotransformations.

#### Production of Carboligases

Wild-type KPHMT and variants and wild-type YfaU as maltose-binding protein fusion construct (MBP-YfaU) and its variant were expressed and purified as described in our previous publications.^[9b,14]^

#### Activity of PmaLAAD

The activity of *Pma*LAAD was measured as described by Busto et al.^[12a]^ For a better comparison of the enzymatic activity of the lyophilized cell preparation, the activity for the oxidation of l-phenylalanine (**1 n**) was determined by measuring the initial rate by HPLC. The assay mixture contained **1 n**(10 mM) in phosphate buffer(100 mM, pH 7) at room temperature. Reactions were started by the addition of the *E. coli* cells containing overexpressed *Pma-*LAAD (5 mg). The conversion was determined between 1 and 2.5 min each 30 s. One unit of activity was defined as the amount of catalyst that catalyzed the oxidation of **1 n**. Measurements were performed in triplicates. The activity of *Pma*LAAD used in the reactions was 0.29 U mL^-1^, corresponding to 0.06 U mg^-1^ cells.

### Synthesis of 2-Substituted 3-Hydroxycarboxylic Acid Derivatives (5)

Representative preparative transformation. **(*S*)-2-** **(hydroxymethyl)-3-methylbutanoic acid** (**5 c**). l-Leucine (**1c**, 66mg, 0.5mmol, 10mM) was added to a suspension of *Pma*LAAD (25 U, 500 mg lyophilized cells), NiCl2 (0.6 mM), and MBP-YfaU W23V (100 mg of lyophilized powder), formaldehyde (563 μL of a 13.4 M stock solution, 7.5 mmol, 150 mM) adjusted to 50 mL total volume with borate buffer (50mM, pH 7.5) in a 100 mL Erlenmeyer flask with plastic screw caps. The flask was shaken for 24 h at 300 rpm at room temperature. The reaction mixture (50 mL) was centrifuged and after discarding the pellet, hydrogen peroxide (3 mL of an 8.8 M commercial solution) was added to the reaction mixture and stirred until completion. Then, catalase from bovine liver (50 mg) dissolved in 1 mL phosphate buffer (10 mM, pH 7.0) was added. The reaction mixture was diluted with methanol (20 mL) and filtered over Celite^®^. After the solvent was evaporated, the aqueous solution was saturated with NaCl and washed with hexane (3 ×50 mL). Then, the pH was adjusted to 1 with HCl (5 N) and the mixture was extracted with Et_2_O (3 × 50 mL). The organic phase was dried over MgSO_4_ and the solvent was removed. In case impurities were detected, the residue was taken up in Et_2_O, washed with a saturated sodium hydrogencarbonate solution (50 mL) and after acidifying the aqueous phase with HCl (pH 1, 5 N) it was extracted again with Et_2_O (3 ×50 mL) and dried over MgSO_4_. The final product was obtained after evaporating the solvent, and no further purification was conducted, therefore the products may contain some minor impurities from the freeze dried *E. coli* cell preparation, as detected in the NMR spectra. Yield: (*S*)-**5 c** (39 mg, 59%, oil), 97% ee, The ee was determined by HPLC analysis on (*S*)-**6 c** derivative (CHIRALPAK^®^ ID 4.6×250 mm column, 5 μm, flow rate 0.7mLmin^-1^ at 20 °C and UV detection (254 nm), isocratic elution hexane:*i*PrOH 75:25; t_R_ (*S*)=17.8min;t_R_ (*R*)= 20.1 min). [α]^20^D = + 5.4 *(c* 5.5, CHCl_3_) (lit.:^[10j]^ [α]^20^D = -5.4 (*c* 4.8, CHCl3) (*R*). The NMR spectra of this product were indistinguishable from (*R*)-**5 c**.

#### (*R*)-2-(hydroxymethyl)-3-methylbutanoic acid (5 c)

The title compound was prepared using KPHMT I202 A variant (50 mg, from a glycerol stock), 0.6 mM of CoCl_2_, and 0.5 mL of an 8.8 M commercial hydrogen peroxide solution for the oxidative decarboxylation reaction, following the procedure described for (*S*)-**5 c**. The product (*R*)-**5 c** was obtained as an oil (31 mg, 47%), 95% ee The ee was determined by HPLC analysis on (*S*)-**6 c** derivative (CHIRALPAK^®^ ID 4.6×250 mm column, 5 μm, flow rate 0.7mLmin^-1^ at 20 °C and UV detection (254 nm), isocratic elution hexane:*i*PrOH 75:25; tR (*S*)=17.8min;tR (*R*)= 20.1 min). [α]^20^D = -4.0 (*c* 2.0, CHCl_3_), (lit.:^[10j]^ [α]^20^D = -5.4 (*c* 4.8, CHCl3)). The NMR spectra of this product agreed with those reported in the literature.^[9b]^ NMR of *S-* **5 c** and *R-* **5 c**:^1^H NMR(CDCl3,400MHz):*δ*=3.91–3.79 (m,2H),2.46–2.41(m, 1H), 2.07–2.00(m, 1H), 0.99 (d, *J*=4.5Hz, 3H), 0.98(d, *J*= 4.5 Hz, 3H). ^13^C NMR (CDCl3, 101 MHz): *δ*=179.9, 61.6, 54.2, 27.8, 20.7, 20.3.

#### 3-Hydroxy-2,2-dimethylpropanoic acid (5 a)

The title compound was prepared using wild-type KPHMT and following the procedure described for **5 a** (substrates: **1 a** (59 mg, 0.5 mmol), formaldehyde (563 μL of a 13.4 M stock solution, 7.5 mmol)).

The product **5 a** was obtained as a solid (30 mg, 51%). The NMR spectra of this product agreed with those reported in the literature.^[18] 1^H NMR (CDCl_3_, 400 MHz): *δ*=3.60 (s, 2H) 1.23 (s, 6H). ^13^C NMR (CDCl3, 101 MHz): *δ*=182.9, 69.5, 44.2 22.1 ppm.

#### (*S*)-3-Hydroxy-2-methylpropanoic acid ((*S*)-5 d)

The title compound was prepared using MBP-YfaU W23V following the procedure described for (*S*)-**5 d** (substrates: **1 d** (52 mg, 0.5 mmol), formaldehyde (563 μL of a 13.4 M stock solution, 7.5 mmol)). The product (*S*)-**5 d** was obtained as an oil (30 mg, 53%), 98% ee. The ee was determined by HPLC analysis on (*S*)-**6 d** derivative (CHIRALCEL^®^ ID 4.6×250 mm column, 5 μm, flow rate 0.7mLmin^-1^ at 20 °C and UV detection (254 nm), isocratic elution hexane:*i*PrOH 75:25; t_R_ (*S*)= 15.7 min; tR (*R*)=17.0 min). [α]^20^D = _11.7 (*c* 1.2, EtOH) (lit.:^[19]^ [α]^20^D = -11.6 (c 1.0, EtOH) *(R)).* The NMR spectra of this product were indistinguishable from those of (*R*)-**5 d**.

#### (*R*)-3-Hydroxy-2-methylpropanoic acid ((*R*)-5 d)

The title compound was prepared using KPHMT I212A following the procedure described for (*R*)-**5 d** (substrates: **1 d** (52 mg, 0.5 mmol), formaldehyde (563 μL of a 13.4 M stock solution, 7.5 mmol)). The product (*R*)-**5 d** was obtained as an oil (11 mg, 22%), 98% ee. The ee was determined by HPLC analysis on (*S*)-**6 d** derivative (CHIRALCEL^®^ ID 4.6×250 mm column, 5 μm, flow rate 0.7mLmin^-1^ at 20°C and UV detection (254 nm), isocratic elution hexane:*i*PrOH 75:25; t_R_ (*S*)= 15.7 min; t_R_ (*R*) = 17.0 min). [α]^20^D = -11.0 *(c* 0.5, EtOH) (lit.:^[19]^ [α]^20^D = -11.6 (*c* 1.0, EtOH) (*R*)). The NMR spectra of this product agreed with those reported in the literature.^[19]^ NMR of*S*-**5 d** and *R-* **5 d**: ^1^H NMR (400 MHz, CDCl3) δ=3.75 (d, *J =* 6.0 Hz, 2H), 2.75 -2.68 (m, 1H), 1.21 (d, *J=* 7.3 Hz, 3H) ppm. ^13^C NMR (101 MHz, CDCl3): *δ*=180.7, 64.5, 41.6, 13.3 ppm.

**(*S*)-2-(Hydroxymethyl)-2-methylbutanoic acid ((*S*)-5 e)**. The title compound was prepared using KPHMT I212A following the procedure described for (*S*)-**5 e** (substrates: **1 e** (66 mg, 0.5 mmol), formaldehyde (563 μL of a 13.4 M stock solution, 7.5 mmol)). The product (*S*)-**5 e** was obtained as an oil (49 mg, 45%), 98% *ee.* The ee was determined by HPLC analysis on (*S*)-**6 e** derivative (CHIRALPAK^®^ ID 4.6×250 mm column, 5 μm, flow rate 0.7mLmin^-1^ at 20°C and UV detection (254 nm), isocratic elution hexane:*i*PrOH 75:25; t_R_ (*S*)= 45.8 min; tR (*R*)=48.4 min). [α]^20^D= _4.0 (*c* 0.7, CHCl3) (lit.:^[10j]^ [α]^20^D = -4.8 (*c* 4.0, CHCl_3_) (*R*)). The NMR spectra of this product agreed with those reported in the literature.^[10j] 1^H NMR (CDCl3, 400 MHz): *δ*=3.76–3.73 (m, 1H), 3.55–3.52 (m, 1H), 1.72–1.58 (m, 2 H), 1.2 (s, 3H), 0.92 (t, *J*=7.5 Hz, 3H) ppm. ^13^C NMR (CDCl3, 101 MHz): *δ*=182.7, 67.8, 48.0, 28.5, 19.0, 8.7 ppm.

#### (*S*)-2-(Hydroxymethyl)butanoic acid ((*S*)-5 g)

The title compound was prepared using MBP-YfaU W23V following the procedure described for (*S*)-**5 g** (substrates: **1 g** (59 mg, 0.5 mmol), formaldehyde (563 μL of a 13.4 M stock solution, 7.5 mmol)). The product (*S*)-**5 g** was obtained as an oil (21 mg, 70%), 96% ee. The ee was determined by HPLC analysis on (*S*)-**6 g** derivative (CHIRALPAK^®^ ID 4.6×250 mm column, 5 μm, flow rate 0.7mLmin^-1^ at 20°C and UV detection (254 nm), isocratic elution hexane:*i*PrOH 75:25; t_R_ (*S*)= 13.8 min; tR (*R*)=15.8 min). [α]^20^D= _3.5 (*c* 0.8, CHCl3) (lit.:^[10j]^ [α]^20^D = -4.8 (*c* 4.0, CHCl_3_) (*R*)). The NMR spectra of this product were indistinguishable from those of (*R*)-**5 g**.

#### (*R*)-2-(Hydroxymethyl)butanoic acid ((*R*)-5 g)

The title compound was prepared using wild-type KPHMT following the procedure described for (*R*)-**5 g** (substrates: **1 g** (59 mg, 0.5 mmol), formaldehyde (563 μL of a 13.4 M stock solution, 7.5 mmol)). The product (*R*)-**5 g** was obtained as an oil (12 mg, 40%), 97% *ee.* The ee was determined by HPLC analysis on (*R*)-**6 g** derivative (CHIRALPAK^®^ ID 4.6×250 mm column, 5 μm, flow rate 0.7mLmin^-1^ at 20°C and UV detection (254 nm), isocratic elution hexane:*i*PrOH 75:25; tR (*S*)= 13.8 min; t_R_ (*R*) = 15.8 min). [α]^20^D = -5.0 (*c* 0.7, CHCl_3_) (lit.:^[10j]^ [α]^20^D = -4.8 (c 4.0, CHCl_3_) (*R*)). The NMR spectra of this product agreed with those reported in the literature.^[20]^ NMR of *S-* **5 g** and *R-* **5 g**: ^1^H NMR (CDCl3, 400 MHz): *δ*= 3.82–3.80 (2H, m), 2.60–2.53 (1 H, m), 1.78–1.60 (2H, m), 1.00 (t, *J*=7.5 Hz, 3H) ppm. ^13^C NMR (CDCl3, 101 MHz): *δ*= 179.7, 62.7, 48.7, 21.6, 11.8.

#### (*S*)-2-(Hydroxymethyl)pentanoic acid ((*S*)-5 h)

The title compound was prepared using MBP-YfaU W23V following the procedure described for (*S*)-**5 h** (substrates: **1 h** (66 mg, 0.5 mmol), formaldehyde (563 μL of a 13.4 M stock solution, 7.5 mmol)). The product (*S*)-**5 h** was obtained as an oil (52 mg, 79%), 97% *ee.* The ee was determined by HPLC analysis on (*S*)-**6 h** derivative (CHIRALPAK^®^ ID 4.6×250 mm column, 5 μm, flow rate 0.7mLmin^-1^ at 20°C and UV detection (254 nm), isocratic elution hexane:*i*PrOH 75:25; t_R_ (*S*)= 12.7 min; tR (*R*)=14.4 min). [α]^20^D= _3.1 (*c* 1.1, CHCl3) (lit.:^[10j]^ [α]^20^D = -3.0 (*c* 10, CHCl_3_) (*R*)). The NMR spectra of this product were indistinguishable from those of (*R*)-**5 h**.

#### (*R*)-2-(Hydroxymethyl)pentanoic acid ((*R*)-5 h)

The title compound was prepared using KPHMT I212A following the procedure described for (*R*)-**5 h** (substrates: **1 h** (66 mg, 0.5 mmol), formaldehyde (563 μL of a 13.4 M stock solution, 7.5 mmol)). The product (*R*)-**5 h** was obtained as an oil (25 mg, 38%), 92% *ee.* The ee was determined by HPLC analysis on (*R*)-**6 h** derivative (CHIRALPAK^®^ ID 4.6×250 mm column, 5 μm, flow rate 0.7mLmin^-1^ at 20°C and UV detection (254 nm), isocratic elution hexane:*i*PrOH 75:25; t_R_ (*S*)= 12.7 min; t_R_ (*R*) = 14.4 min). [α]^20^D = -4.2 (*c* 0.7, CHCl_3_) (lit.:^[10j]^ [α]^20^D = -3.0 (*c* 10, CHCl_3_)(*R*)). The NMR spectra of this product agreed with those reported in the literature.^[9b]^ NMR of *S*-**5 h** and *R-* **5 h**: ^1^H NMR (CDCl3, 400 MHz): *δ*=δ 3.80–3.73 (m, 2H), 2.67–2.60 (m, 1H), 1.70–1.65 (m, 1H), 1.58–1.49 (m, 1H), 1.44–1.38 (m, 2H), 0.94 (t, *J*=7.3 Hz, 3H).^13^C NMR (CDCl3, 101 MHz): *δ*=180.4, 63.9, 47.4, 30.5, 20.5, 14.1.

#### (*S*)-2-Benzyl-3-hydroxypropanoic acid ((*S*)-5 i)

The title compound was prepared using MBP-YfaU W23V following the procedure described for (*S*)-**5 i** (substrates: **1 i** (90 mg, 0.5 mmol), formaldehyde (563 μL of a 13.4 M stock solution, 7.5 mmol)). The product (*S*)-**5 i** was obtained as a solid (56 mg, 62%), 95% *ee.* The ee was determined by HPLC analysis on (*S*)-**6 i** derivative (CHIRALPAK^®^ ID 4.6×250 mm column, 5 μm, flow rate 0.7mLmin^-1^ at 20°C and UV detection (254 nm), isocratic elution hexane:*i*PrOH 75:25; t_R_ (*S*)= 26.3 min; t_R_ (*R*) = 25.5 min). [α]^20^D = -10.4 (*c* 1.5, CHCl_3_) (lit.:^[10j]^ [α]^20^_D_= _12.5 (*c* 2.0, CHCl_3_) (*R*)). The NMR spectra of this product were indistinguishable from those of (*R*)-**5 i**.

#### (*R*)-2-Benzyl-3-hydroxypropanoic acid ((*R*)-5 i)

The title compound was prepared using KPHMT I212A following the procedure described for (*R*)-**5 i** (substrates: **1 i** (90 mg, 0.5mmol), formaldehyde (563μL of a 13.4M stock solution, 7.5 mmol)). The product (*R*)-**5 i** was obtained as a solid (48 mg, 43%), 91% *ee.* The ee was determined by HPLC analysis on (*R*)-**6 i** derivative (CHIRALPAK^®^ ID 4.6×250mm column, 5 μm, flow rate 0.7mLmin^-1^ at 20°C and UV detection (254 nm), isocratic elution hexane:*i*PrOH 75:25; tR (*S*)= 26.3min; tR (*R*)=25.5min).[α]^20^D=_12.7 (*c* 3.0, CHCl3) (lit.:^[10j]^ [α]^20^_D_= _12.5 (*c* 2.0, CHCl_3_) (*R*)). The NMR spectra of this product agreed with those reported in the literature.^[10j]^ NMR of (*S*)-**5 i** and (*R*)*-* **5 i**: ^1^H NMR (400 MHz; CDCl3) *δ*= 7.32–7.20 (m, 5H), 3.80–3.70 (m, 2H), 3.09–3.06 (m, 1H), 2.90–2.82 (m, 2H) ppm. ^13^C NMR (CDCl3, 101 MHz) *δ*= 179.7,138.4,129.1,128.7,126.8,62.1,49.0,34.2ppm.

## Supplementary Material

Supporting information for this article is available on the WWW under https://doi.org/10.1002/adsc.202100145

Supporting Information

## Figures and Tables

**Table 1:** Aldol product (*S*)-**3 c** formation by employing the enzymatic cascade reaction with varying concentrations of **1 c** and formaldehyde. Conditions: lyophilized whole cells *Pma-* LAAD (1.1 U), MBP-YfaU-W23V (2 mg), NiCl2 (0.6 mM), MilliQ water (1 mL), rt, horizontal shaking, 24 h

Entry	Formaldehyde[mM]	1c[mM]	(*S*)-3c[mM]	(*S*)-3c[%]
1	30	10	3.6	36
2	50	50	10.0	20
3	75	25	10.8	43
4	75	50	9.5	19
5	100	50	12.0	24
6	150	10	7.8	78
7	150	25	15.3	61
8	150	50	18.5	37
9	150	75	24.0	32
10	250	50	15.5	31
11	500	50	7.0	14

**Table 2:** Carboligase screening in the deamination/aldol addition cascade reaction with varying substrates.^[a]^

Entry	Carboligase	Conversion of amino acid **1** [%]^[b]^	2-Oxoacid **2** [%]^[c]^	Aldol Product **3** [%]^[d]^	Aldol Product **3**
1	wild-type KPHMT	58	22	36(**3a**)	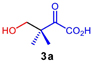
2	KPHMT-I202A	54	20	34(**3a**)	3a
3	KPHMT-I212A	54	30	24(**3a**)	
4	wild-type MBP-YfaU	53	50	3(**3a**)	
5	MBP-YfaU-W23V	47	45	2(**3a**)	
6	wild-type KPHMT	>99	79	21(*R*-**3c**)	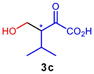
7	KPHMT-I202A	>99	22	78(*R*-**3c**)	3c
8	KPHMT-I212A	>99	91	9(*R*-**3c**)	
9	wild-type MBP-YfaU	>99	98	2(*S*-**3c**)	
10	MBP-YfaU-W23V	>99	20	80(*S*-**3c**)	
11	wild-type KPHMT	>99	0	75 (*R*-**3d**)	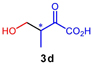
12	KPHMT-I202A	>99	0	54 (*R*-**3d**)	3d
13	KPHMT-I212A	>99	0	79 (*R*-**3d**)	
14	wild-type MBP-YfaU	>99	0	98 (*S*-**3d**)	
15	MBP-YfaU-W23V	>99	0	96 (*S*-**3d**)	
16	wild-type KPHMT	>99	11	89 (**3e**)	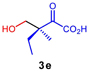
17	KPHMT-I202A	>99	24	76 (**3e**)	3e
18	KPHMT-I212A	90	20	70 (**3e**)	
19	wild-type MBP-YfaU	>99	85	15 (**3e**)^[e]^	
20	MBP-YfaU-W23V	85	71	14 (**3e**)^[e]^	
21	wild-type KPHMT	66	39	22 (**3f**)^[e]^	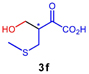
22	KPHMT-I202A	64	10	22 (**3f**)^[e]^	3f
23	KPHMT-I212A	59	12	41 (**3f**)^[e]^	
24	wild-type MBP-YfaU	58	16	36 (**3f**)^[e]^	
25	MBP-YfaU-W23V	64	9	49 (**3f**)^[e]^	
26	wild-type KPHMT	>99	6	94 (*R*-**3g**)	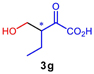
27	KPHMT-I202A	>99	4	55 (*R*-**3g**)	3g
28	KPHMT-I212A	>99	10	90 (*R*-**3g**)	
29	wild-type MBP-YfaU	>99	57	43 (*S*-**3g**)	
30	MBP-YfaU-W23V	>99	4	96 (*S*-**3g**)	
31	wild-type KPHMT	>99	68	27 (*R*-**3h**)	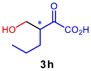
32	KPHMT-I202A	>99	4	60 (*R*-**3h**)	3h
33	KPHMT-I212A	>99	5	89 (*R*-**3h**)	
34	wild-type MBP-YfaU	95	47	48 (*S*-**3h**)	
35	MBP-YfaU-W23V	97	3	94 (*S*-**3h**)	
36	wild-type KPHMT	>99	94	6 (*R*-**3i**)	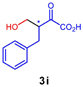
37	KPHMT-I202A	>99	75	25 (*R*-**3i**)	3i
38	KPHMT-I212A	>99	10	90 (*R*-**3i**)	
39	wild-type MBP-YfaU	>99	91	9 (*S*-**3i**)	
40	MBP-YfaU-W23V	>99	7	93 (*S*-**3i**)	

[a] Conditions: lyophilized whole cells *Pma*LAAD (1.1 U), MBP-YfaU-W23V (2 mg) or KPHMT-I202A (1 mg), NiCl2 or CoCl2 (0.6 mM), respectively, MilliQ water (1 mL), rt, horizontal shaking, 24 h.

[b] Conversion = **2** [%] +**3** [%].

[c] Percentage of unreacted 2-oxoacid, not converted in the aldol addition to formaldehyde, determined by HPLC.

[d] Formation of aldol adduct determined by HPLC.

[e] Configuration not determined.
