# Supplementary material for: Chemoenzymatic Production of Enantiocomplementary 2-Substituted 3-Hydroxycarboxylic Acids from L-α-Amino Acids
Source: Adv Synth Catal. Author manuscript; Available in PMC 2021 Jul 16. (PMC7611260; doi:10.1002/adsc.202100145)
Supplement: Supporting Information [file EMS129396-supplement-Supporting_Information.pdf]

Advanced Synthesis & Catalysis

Supporting Information

Chemoenzymatic Production of Enantiocomplementary 2-Substituted 3-Hydroxycarboxylic Acids from L-α-Amino Acids

Mathias Pickl,* Roser Marín-Valls, Jesús Joglar, Jordi Bujons, and Pere Clapés*© 2021 The Authors. Advanced Synthesis & Catalysis published by Wiley-VCH GmbH. This is an open access article under the terms of the Creative Commons Attribution License, which permits use, distribution and reproduction in any medium, provided the original work is properly cited.

Supporting Information

Chemoenzymatic production of enantiocomplementary 2-substituted 3-hydroxycarboxylic acids from L-α-amino acids

Mathias Pickl,[a,b*] Roser Marín-Valls,[a] Jesús Joglar,[a] Jordi Bujons,[a] Pere Clapés.[a*]

[a]Department of Chemical Biology. Institute for Advanced Chemistry of Catalonia (IQAC−CSIC), Jordi Girona 18-26, 08034 Barcelona, Spain. Phone +34934006127, Fax: +34932045904, E-mail: pere.clapes@iqac.csic.es.

[b]Present address: Department of Chemistry, University of Graz, Heinrichstrasse 28, 8010 Graz, Austria., Email: mathias.pickl@uni-graz.at

0 Table of contents

0 Table of contents 2

1 Optimization Studies... 3

1.1 Endpoint study of *Pma*LAAD catalyzed oxidative deamination of amino acids (Figure S1). 3

1.2 Initial conditions of one-pot cascade synthesis of (*S*)- and (*R*)-3-(hydroxymethyl)-4-methyl-2-oxopentanoic Acid ((*S*)-3c and (*R*)-3c) for optimization studies (Figure S2).. 4

1.3 Carboligase loading effect on the enzymatic cascade synthesis of (*S*)-(hydroxymethyl)-4-methyl-2-oxopentanoic acid ((*S*)-3c) and 4-hydroxy-3,3-dimethyl-2-oxobutanoic acid (3a) (Figure S3). 4

1.4 Optimized cascade conditions for the synthesis of (*S*)- and (*R*)-3-(hydroxymethyl)-4-methyl-2-oxopentanoic acid ((*S*)-3c and (*R*)-3c). 5

1.5 Two-step procedure of *Pma*LAAD/carboligase for the synthesis of (*S*)-3-(hydroxymethyl)-4-methyl-2-oxopentanoic acid ((*S*)-3c) (Figure S4)... 5

1.6 Solvent effect on the enzymatic cascade synthesis of (*S*)-3-(hydroxymethyl)-4-methyl-2-oxopentanoic acid ((*S*)-3c) (Figure S5).. 6

1.7 Inhibition study with ammonia present (Figure S6). 7

1.8 Effect of amino acid deaminase *Pma*LAAD loading in one-pot cascade synthesis of (*R*)-3-(hydroxymethyl)-4-methyl-2-oxopentanoic acid ((*R*)-3c) under optimized conditions (Figure S7)....... 7

2 Additional substrates tested, which did not provide aldol product formation (Figure S8). 8

3 Analytical data of HPLC monitoring reactions.. 9

3.1 HPLC retention time (Table S1) of derivatized of 2-oxoacids 2 and 3-substituted 4-hydroxy-2-oxoacids 3. 9

3.2 Reaction monitoring chromatograms. HPLC traces of derivatized cascade products of the reactions after 24 h. 10

4 Chiral HPLC 12

5 Chiral HPLC chromatograms 13

5.1 Compound 6c... 13

5.2 Compound 6d 14

5.3 Compound 6e 15

5.4 Compound 6g... 16

5.5 Compound 6h 17

5.6 Compound 6i 18

6 NMR spectra. 20

1 Optimization Studies.

1.1 Endpoint study of *Pma*LAAD catalyzed oxidative deamination of amino acids (Figure S1).

The amino acid (50 mM) was added to a suspension of *Pma*LAAD (1.1 U, 20 mg lyophilized cells), adjusted to 1 mL total volume with phosphate buffer (100 mM, pH 7) in a 6 mL glass vial with plastic screw caps. The vial was placed in a horizontal shaker for 24 h at 1000 rpm and room temperature. An aliquot was withdrawn and derivatized before HPLC measurements.

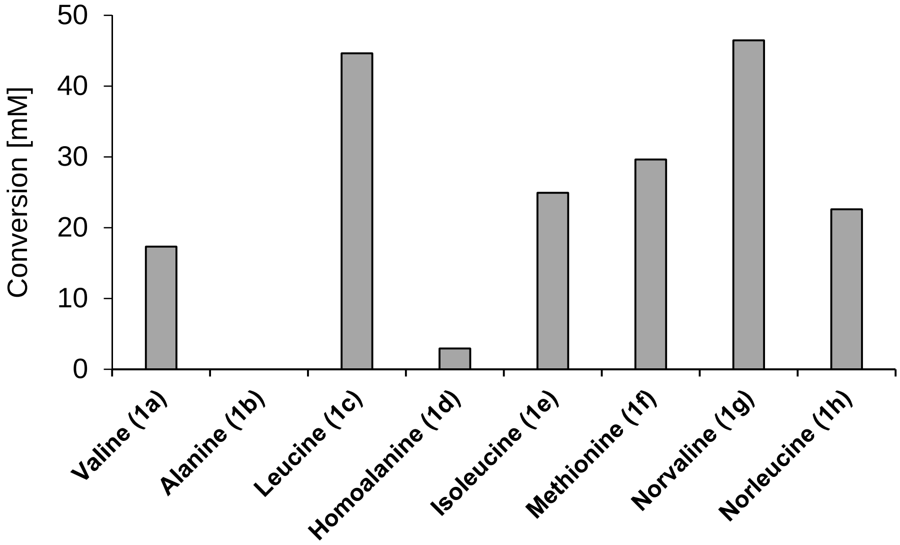

Figure S1. Endpoint study of selected L-amino acid substrates **1** for oxidative deamination catalyzed by *Pma*LAAD. Conditions: 50 mM substrate, phosphate buffer (100 mM, pH 7), 24 h, room temperature, horizontal shaking.

1.2 Initial conditions of one-pot cascade synthesis of (*S*)- and (*R*)-3-(hydroxymethyl)-4-methyl-2-oxopentanoic acid ((*S*)-3c and (*R*)-3c) for optimization studies (Figure S2).

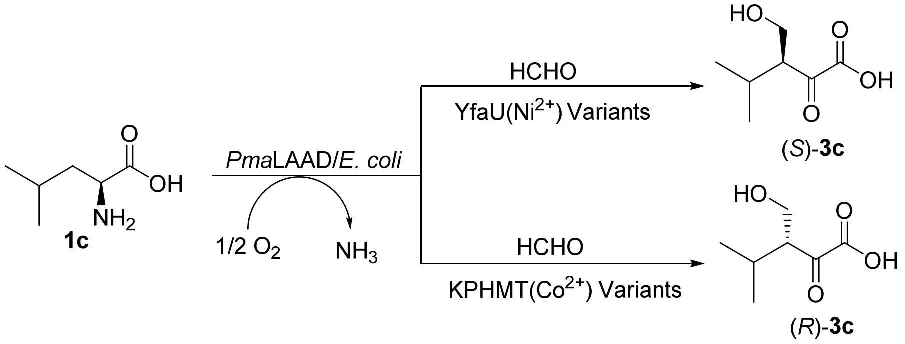

Figure S2. One-pot simultaneous multi-enzyme system for the synthesis of enantiomerically pure (*R*)- or (*S*)-3-(hydroxymethyl)-4-methyl-2-oxopentanoic acid starting from **1c** and formaldehyde.

L-Leucine (**1c**, 50 mM) was added to a suspension of *Pma*LAAD (1.1 U, 20 mg lyophilized whole cells), containing $NiCl_2$ or $CoCl_2$ (0.6 mM) depending on the enzyme, carboligase (1 mg in case of KPHMT from a glycerol stock, or 2 mg in case of MBP-YfaU as a lyophilized powder), and formaldehyde (50 mM) adjusted to 1 mL total volume with MilliQ water in a 6 mL glass vial with plastic screw caps. The vial was placed in a horizontal shaker and shaken for 24 h at 1000 rpm and room temperature. An aliquot was withdrawn and derivatized before HPLC measurements. For optimization studies, the screened parameter was altered.

1.3 Carboligase loading effect on the enzymatic cascade synthesis of (*S*)-(hydroxymethyl)-4-methyl-2-oxopentanoic acid ((*S*)-3c) and 4-hydroxy-3,3-dimethyl-2-oxobutanoic acid (3a) (Figure S3).

Conditions as described above with varying catalyst loading. In case of KPHMT-*wt* **1a** was used as model substrate.

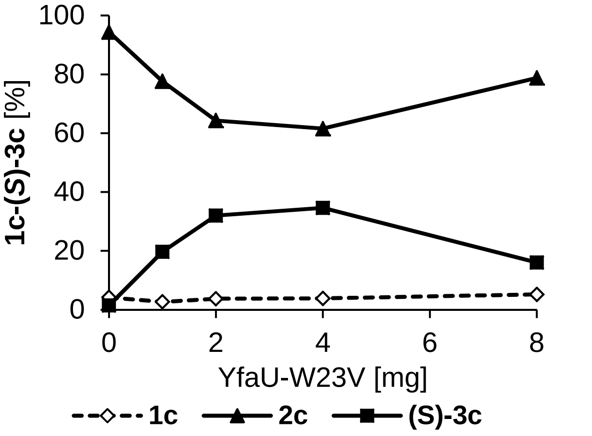

Conditions: 1.1 U *Pma*LAAD, 50 mM **1c**, 50 mM formaldehyde, 1 mL total, MBP-YfaU-W23V, horizontal shaking, rt, 24 h.

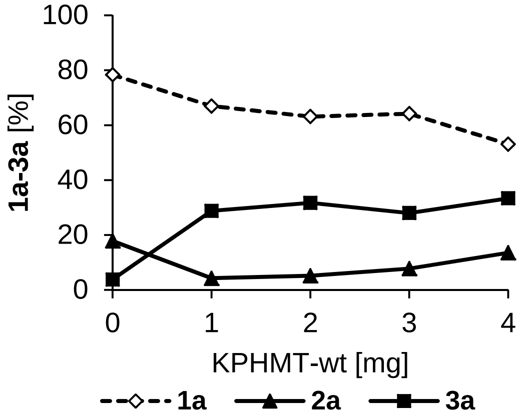

Conditions: 1.1 U *Pma*LAAD, 50 mM **1a**, 50 mM formaldehyde, 1 mL total, KPHMT-*wt*, horizontal shaking, rt, 24 h.

Figure S3. Cascade synthesis of (*S*)-3-(hydroxymethyl)-4-methyl-2-oxopentanoic acid **(3c)** and 4-hydroxy-3,3-dimethyl-2-oxobutanoic acid **(3a)**. Endpoint study with varying catalyst load under equimolar conditions using MBP-YfaU-W23V and **1a** in the case of KPHMT-*wt*.

1.4 Optimized cascade conditions for the synthesis of (*S*)- and (*R*)-3-(hydroxymethyl)-4-methyl-2-oxopentanoic acid ((*S*)-3c and (*R*)-3c).

L-Leucine (**1c**, 10 mM) was added to a suspension of *Pma*LAAD (1.1 U, 20 mg lyophilized whole cells), containing $NiCl_2$ or $CoCl_2$ (0.6 mM) depending on the enzyme, carboligase (1 mg of KPHMT from a glycerol stock, 2 mg of MBP-YfaU as a lyophilized powder, respectively), and formaldehyde (150 mM) adjusted to 1 mL total volume with MilliQ water in a 6 mL glass vial with plastic screw caps. The vial was placed in a horizontal shaker and shaken for 24 h at 1000 rpm and room temperature. An aliquot was withdrawn and derivatized before HPLC measurements.

1.5 Two-step procedure of *Pma*LAAD/carboligase for the synthesis of (*S*)-3-(hydroxymethyl)-4-methyl-2-oxopentanoic acid ((*S*)-3c) (Figure S4).

L-Leucine (**1c**, 50 mM) was added to a suspension of *Pma*LAAD (1.1 U, 20 mg lyophilized whole cells), containing $NiCl_2$ or $CoCl_2$ (0.6 mM) depending on the enzyme, and formaldehyde (50 mM) adjusted to 1 mL total volume with MilliQ water in a 6 mL glass vial with plastic screw caps. The vial was placed in a horizontal shaker at 1000 rpm and room temperature. After 7 h the amino acid was completely consumed, the whole cells were removed, variant MBP-YfaU-W23V was added, and the mixture continued shaken for 14 h. Then, an aliquot was withdrawn and derivatized before HPLC measurements.

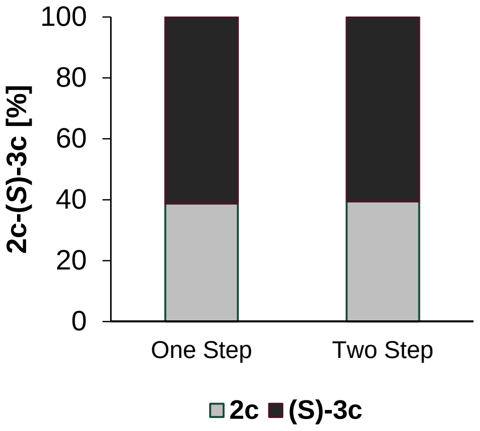

Figure S4. Cascade synthesis of (*S*)-3-(hydroxymethyl)-4-methyl-2-oxopentanoic acid ((*S*)-**3c**). Endpoint study for the comparison of a one-step and a two-step protocol of a one-pot deamination/aldol reaction cascade reaction with substrate **1c**.

1.6 Solvent effect on the enzymatic cascade synthesis of (*S*)-3-(hydroxymethyl)-4-methyl-2-oxopentanoic acid ((*S*)-3c) (Figure S5).

L-Leucine (**1c**, 50 mM) was added to a suspension of *Pma*LAAD (1.1 U, 20 mg lyophilized whole cells), containing $NiCl_2$ (0.6 mM), carboligase (2 mg of MBP-YfaU as a lyophilized powder), and formaldehyde (50 mM) adjusted to 900 µL total volume with MilliQ water in a 6 mL glass vial with plastic screw caps. To this solution, the respective co-solvent (100 µL, 10% v/v total) was added. The vial was placed in a horizontal shaker for 24 h at 1000 rpm and room temperature. Then, an aliquot was withdrawn and derivatized before HPLC measurements.

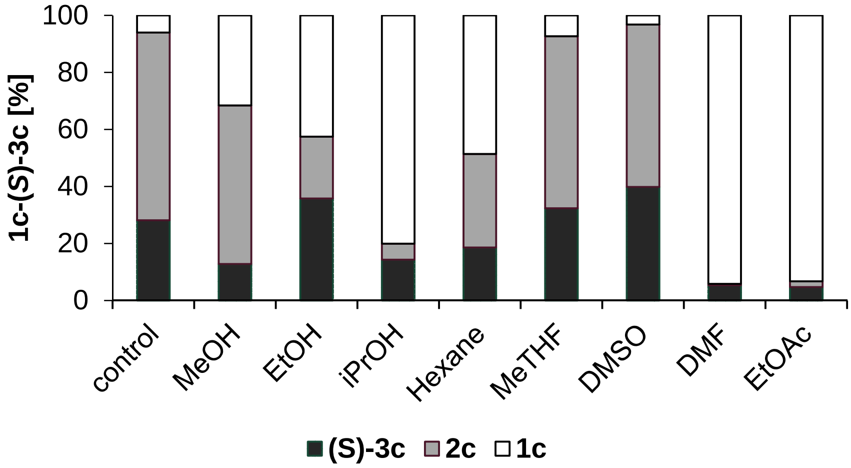

Figure S5. Solvent effect on the enzymatic cascade synthesis of (*S*)-(hydroxymethyl)-4-methyl-2-oxopentanoic acid ((*S*)-**3c**) with substrate **1c** in a simultaneous one-pot cascade of

*Pma*LAAD/MBP-YfaU-W23V with 10% v/v co-solvent loading. (MeTHF: 2-methyltetrahydrofuran)

1.7 Inhibition study with ammonia present (Figure S6).

4-Methyl-2-oxovaleric acid (**2c**, 50 mM) was added to a suspension of $NiCl_2$ (0.6 mM), carboligase (2 mg of MBP-YfaU-W23V as lyophilized powder), and formaldehyde (150 mM) and adjusted to 950 or 975 µL total volume with MilliQ water in an Eppendorf tube. Ammonia (50 µL or 25 µL stock solution, respectively) was added. The tube was placed in a horizontal shaker for 24 h at 1000 rpm and room temperature. Then, an aliquot was withdrawn and derivatized before HPLC measurements.

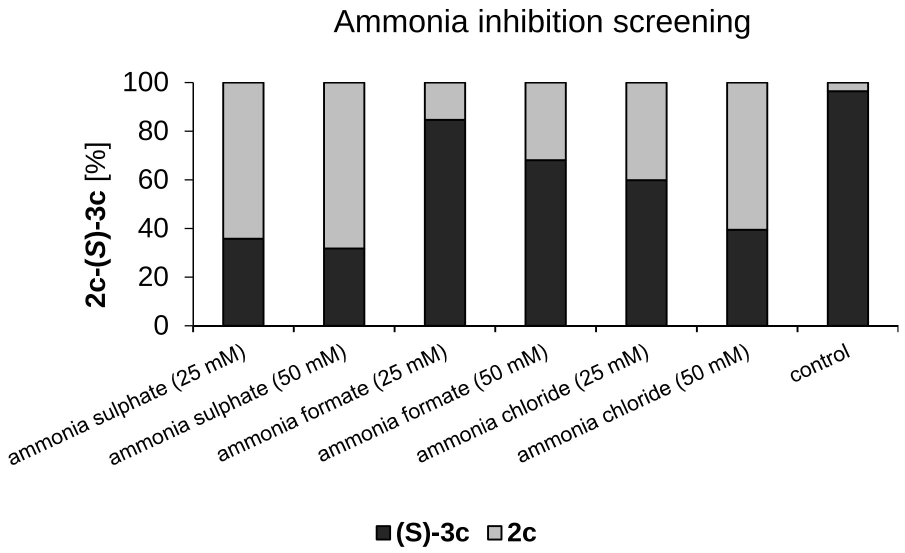

Figure S6. Cascade synthesis of (*S*)-3-(hydroxymethyl)-4-methyl-2-oxopentanoic acid ((*S*)-**3c**). Ammonia inhibition screening with variant MBP-YfaU-W23V. Conditions: 4-methylketovaleric acid (**2c**, 50 mM), formaldehyde (150 mM), MBP-YfaU-W23V (2 mg), $NiCl_2$ (0.6 mM), 24 h, vertical shaking, rt.

1.8 Effect of amino acid deaminase *Pma*LAAD loading in one-pot cascade synthesis of (*R*)-3-(hydroxymethyl)-4-methyl-2-oxopentanoic acid ((*R*)-3c) under optimized conditions (Figure S7).

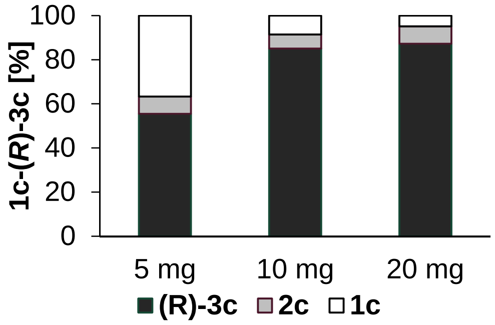

Figure S7. *Pma*LAAD loading effect on one-pot cascade synthesis of (*R*)-3-(hydroxymethyl)-4-methyl-2-oxopentanoic acid ((*R*)-**3c**) with KPHMT-I202A. Conditions: L-leucine (**1c**, 10 mM), formaldehyde (150 mM), $CoCl_2$ (0.6 mM), 24 h, vertical shaking, rt.

2 Additional substrates tested, which did not provide aldol product formation (Figure S8).

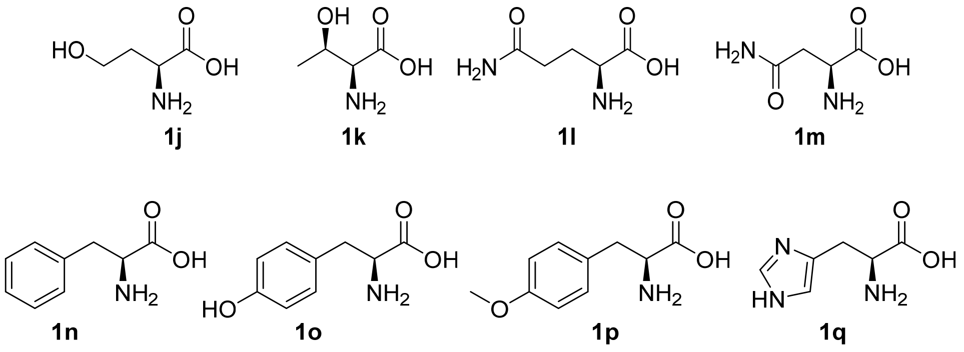

Figure S8. L-α-Amino acids tested for acceptance in deamination/aldol cascade setup.

3 Analytical data of HPLC monitoring reactions.

3.1 HPLC retention time (Table S1) of derivatized 2-oxoacids 2 and 3-substituted 4-hydroxy-2-oxoacids 3.

Table S1. Retention times of *Z*/*E* **2**-oxime and *Z*/*E* **3**-oxime after $BnONH_2$ derivatization

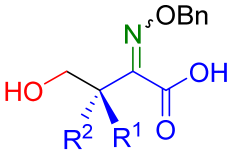

Z/E **3-oxime**

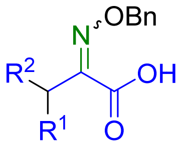

Z/E **2-oxime**

	R^1:	R^2:
**a**:	Me	Me
**c**:	*i*Pr	H
**d**:	Me	H
**e**:	Et	Me
**g**:	Et	H
**h**:	*n*Pr	H
**i**:	Bn	H

4-hydroxy-2-oxoacid	*Z* or *E* **3**-oxime t_R (min)[a]	*Z* or *E* **3**-oxime t_R (min)[a]	2-Oxoacid	*Z or E* **2**-oxime t_R (min)	*Z or E* **2**-oxime t_R (min)
**3a**	17.5 (minor)	18.5 (major)	**2a**	23.3 (major)	24.6 (minor)
**3c**	20.1 (major)	20.9 (minor)	**2c**	24.8 (minor)	25.6 (major)
**3d**	15.9 (minor)	16.3 (major)	**2d**	20.8 (minor)	22.1 (major)
**3e**	20.4	–	**2e**	24.9	–
**3f**	18.6	–	**2f**	24.4	–
**3g**	18.4 (major)	19.4 (minor)	**2g**	23.2 (minor)	24.1 (major)
**3h**	20.7 (major)	21.4 (minor)	**2h**	25.5 (minor)	26.4 (major)
**3i**	22.7	–	**2i**	26.8 (minor)	27.2 (major)

[a]*E*- and *Z*-isomer of **2**-oxime and **3**-oxime are observed as two separate peaks; major and minor refer to the relative size of the corresponding peak. Formaldehyde t_R = 21.6 min.

3.2 Reaction monitoring chromatograms. HPLC traces of derivatized cascade products of the reactions after 24 h.

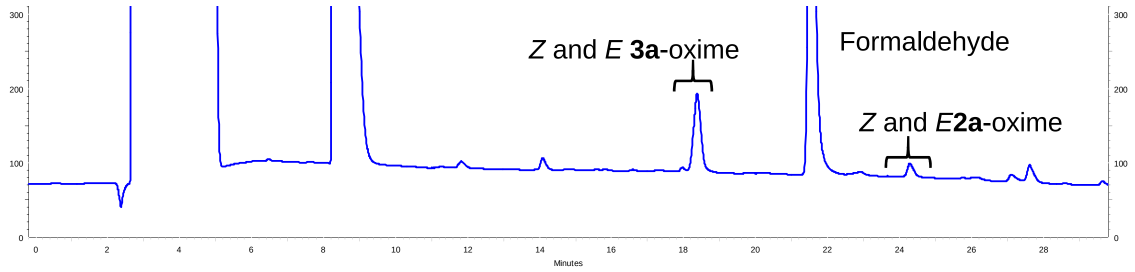

Figure S9. HPLC monitoring trace after 24 h of reaction during the synthesis of **3a**.

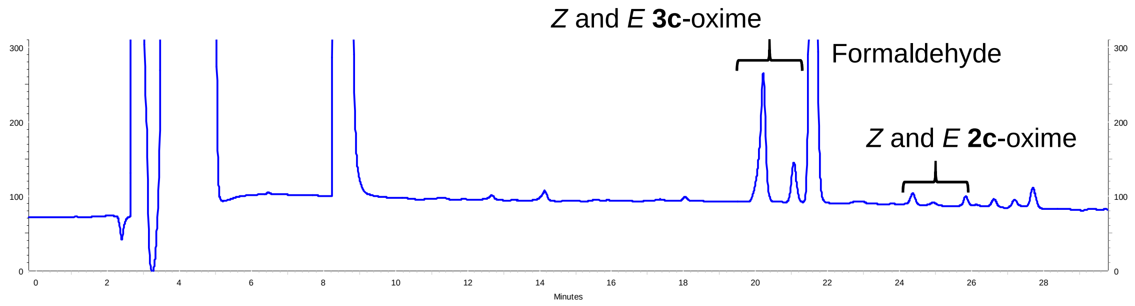

Figure S1. HPLC monitoring trace after 24 h of reaction during the synthesis of **3c**.

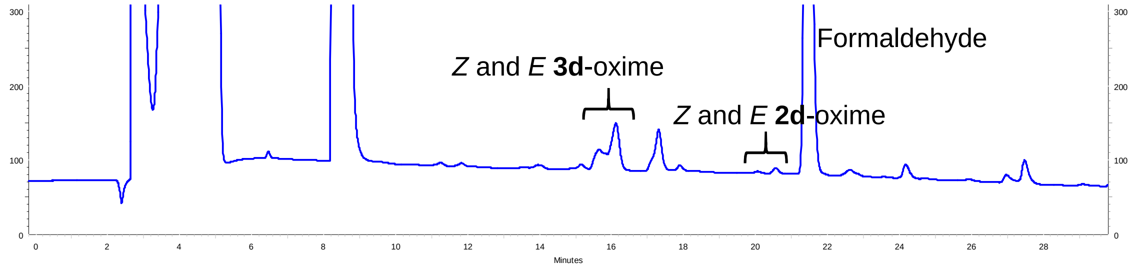

Figure S2. HPLC monitoring trace after 24 h of reaction during the synthesis of **3d**.

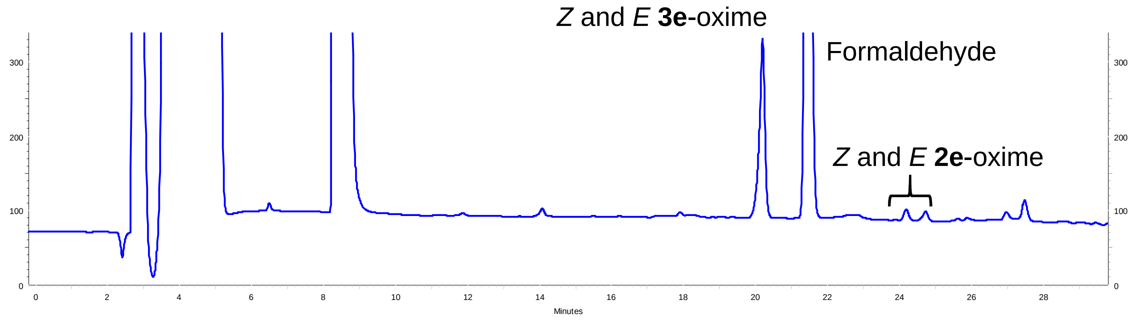

Figure S3. HPLC monitoring trace after 24 h of reaction during the synthesis of **3e**.

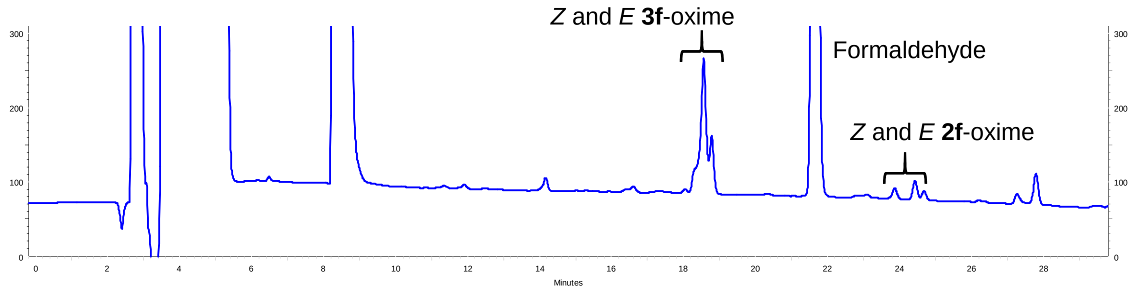

Figure S4. HPLC monitoring trace after 24 h of reaction during the synthesis of **3f**.

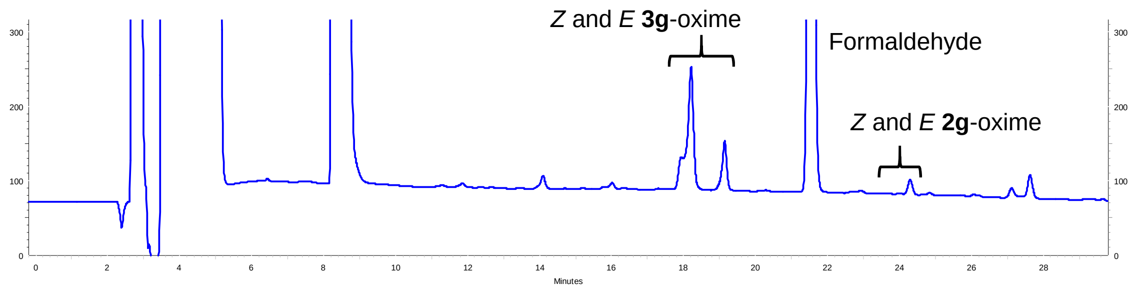

Figure S5. HPLC monitoring trace after 24 h of reaction during the synthesis of **3g**.

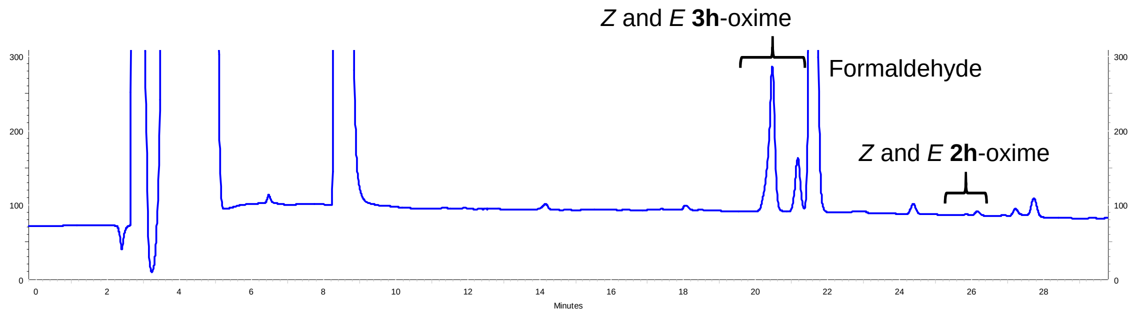

Figure S6. HPLC monitoring trace after 24 h of reaction during the synthesis of **3h**.

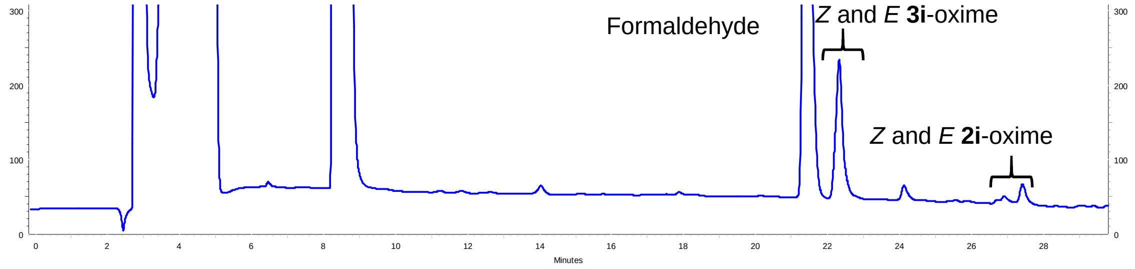

Figure S7. HPLC monitoring trace after 24 h of reaction during the synthesis of **3i**.

Table S2. Summary of optical rotation of isolated chiral 2-hydroxymethyl carboxylic acids **5**.

Decarboxylated Aldol product	Carboligase	Optical rotation	Literature data[a]
(*S*)-**5c**	MBP-YfaU-W23V	$[\alpha]^{20}_{D}$ =.+ 5.4(*c* 5.5, $CHCl_3$)	$[\alpha]^{20}_{D}$ = – 5.4 (*c* 4.8, $CHCl_3$)(*R*)[5]
(*R*)-**5c**	KPHMT-I202A	$[\alpha]^{20}_{D}$ = – 4.0(*c* 2.0, $CHCl_3$)	
(*S*)-**5d**	MBP-YfaU-W23V	$[\alpha]^{20}_{D}$ = +11.7(*c* 1.2, EtOH)	$[\alpha]^{20}_{D}$ = – 11.6 (*c* 1.0, EtOH)(*R*)[6]
(*R*)-**5d**	KPHMT-I212A	$[\alpha]^{20}_{D}$ = – 11.0(*c* 0.5, EtOH)	
(*S*)-**5e**	KPHMT-I212A	$[\alpha]^{20}_{D}$ = + 4.0(*c* 0.7, $CHCl_3$)	$[\alpha]^{20}_{D}$ = – 4.8 (*c* 4.0, $CHCl_3$)(*R*)[5]
(*S*)-**5g**	MBP-YfaU-W23V	$[\alpha]^{20}_{D}$ = + 3.5(*c* 0.8, $CHCl_3$)	$[\alpha]^{20}_{D}$ = – 4.8 (*c* 4.0, $CHCl_3$)(*R*)[5]
(*R*)-**5g**	KPHMT-I212A	$[\alpha]^{20}_{D}$ = – 5.0(*c* 0.7, $CHCl_3$)	
(*S*)-**5h**	MBP-YfaU-W23V	$[\alpha]^{20}_{D}$ = + 3.1(*c* 1.1, $CHCl_3$)	$[\alpha]^{20}_{D}$ = – 3.0 (*c* 10, $CHCl_3$)(*R*)[5]
(*R*)-**5h**	KPHMT-I212A	$[\alpha]^{20}_{D}$ = – 4.2(*c* 0.7, $CHCl_3$)	
(*S*)-**5i**	MBP-YfaU-W23V	$[\alpha]^{20}_{D}$ = – 10.4(*c* 1.5, $CHCl_3$)	$[\alpha]^{20}_{D}$ = +12.5 (*c* 2.0, $CHCl_3$)(*R*)[5]
(*R*)-**5i**	KPHMT-I212A	$[\alpha]^{20}_{D}$ = + 12.7(*c* 3.0, $CHCl_3$)	

[a] *R*-configuration of the reference data obtained from the literature. [Ref.] source of data.

4 Chiral HPLC

Table S3. Retention times of derivatized aldol products **6** on HPLC on chiral stationary phase.

Aldol Product **3**	Aldol Product derivatized **6**	t_R [min] *S*-enantiomer	t_R [min] *R*-enantiomer
**3c**	**6c**	17.8	20.1
**3d**	**6d**	15.7	17.0
**3e**	**6e**	45.8	48.4
**3g**	**6g**	13.8	15.8
**3h**	**6h**	12.7	14.4
**3i**	**6i**	26.3	25.5

5 Chiral HPLC chromatograms

5.1 Compound 6c

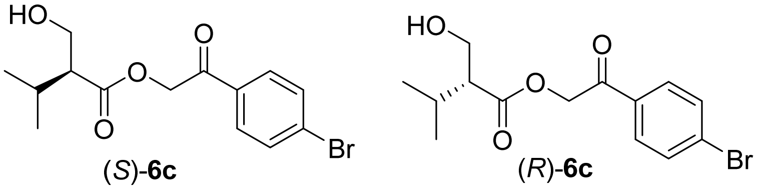

a) *rac*-**6c**

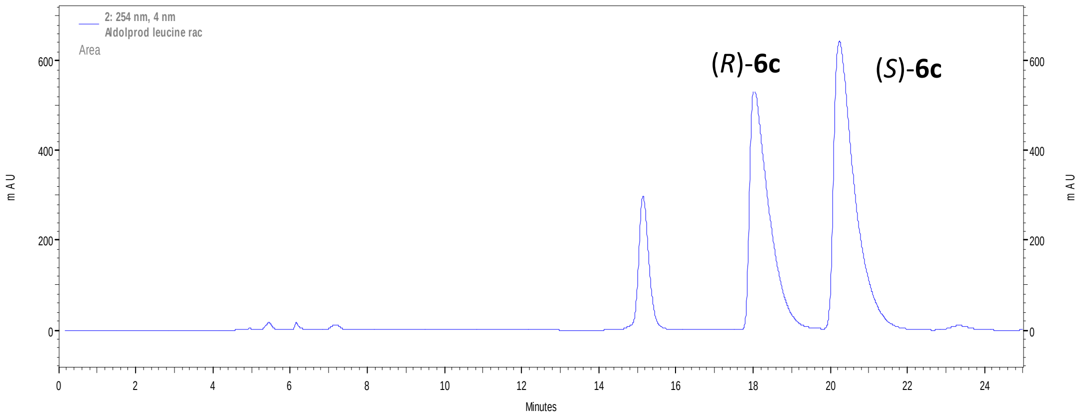

b) (*S*)-**6c**

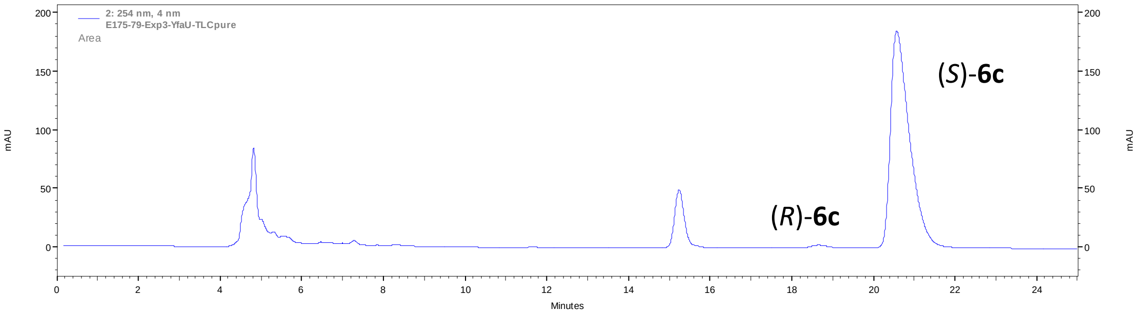

c) (*R*)-**6c**

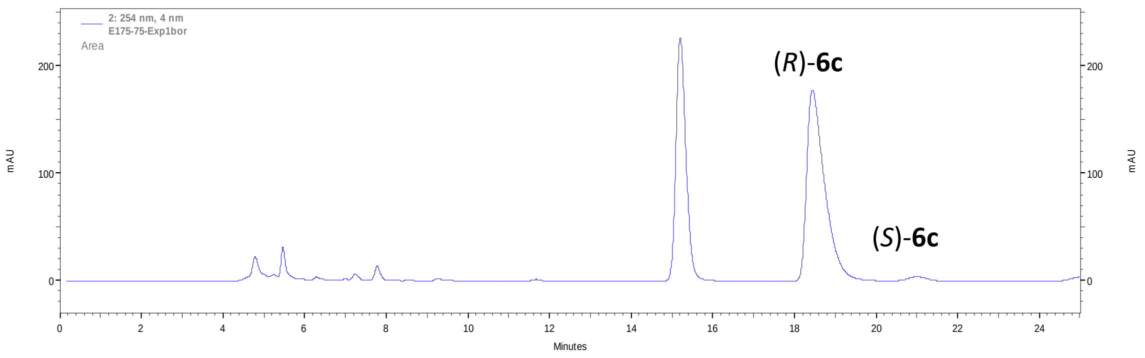

Figure S17. CSP-HPLC analysis chromatogram of *rac*-**6c** (a), derivatized (*S*)-**6c** synthetized with MBP-YfaU-W23V (b), and derivatized (*R*)-**6c** synthetized with KPHMT-I202A (c). Conditions: CHIRALPACK® ID 4.6 x 250 mm column, 5 µm, flow rate 0.7 mL min^{-1} at 20 °C and UV detection (254 nm). Isocratic elution hexane:*i*PrOH 75:25.

5.2 Compound 6d

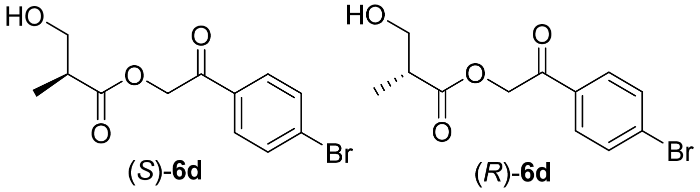

a) *rac*-**6d**

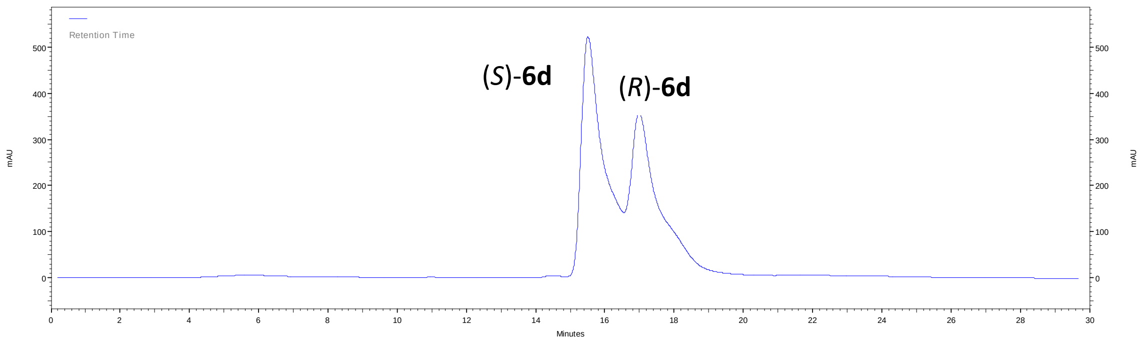

b) (*S*)-**6d**

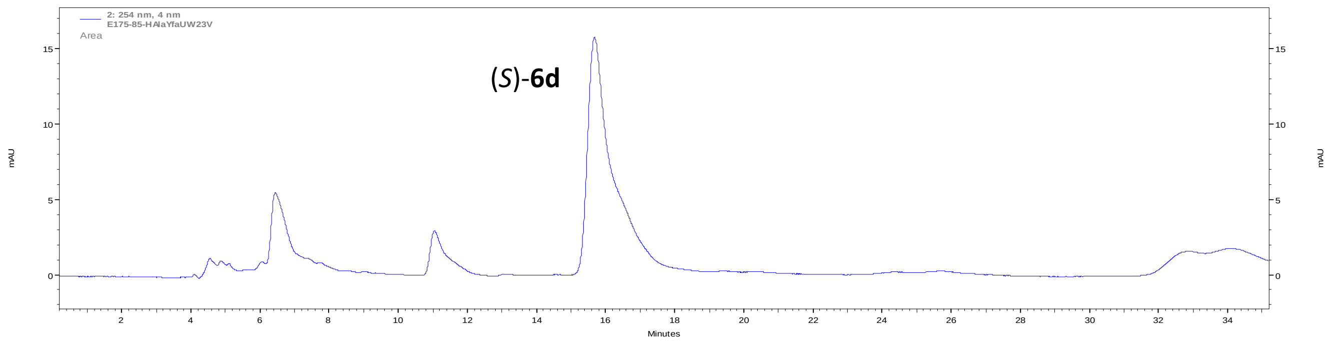

c) (*R*)-**6d**

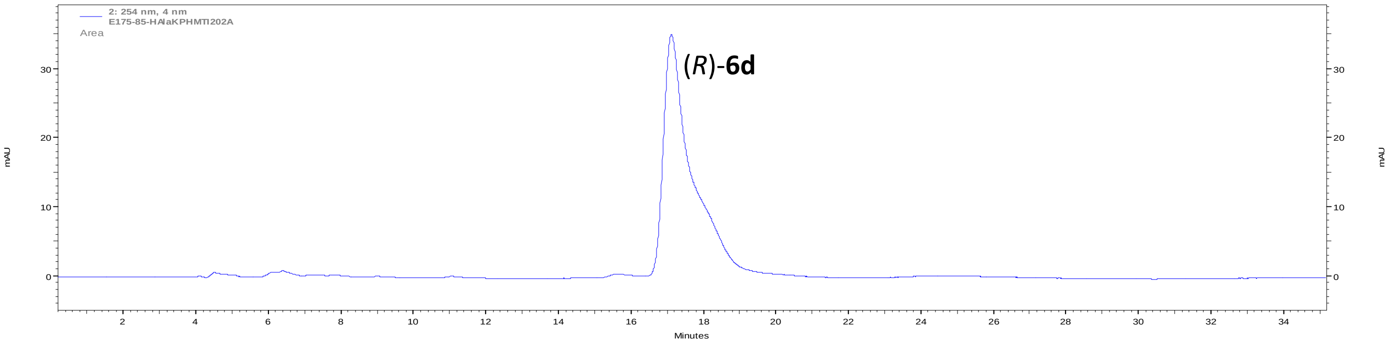

Figure S18. CSP-HPLC analysis chromatogram of of *rac*-**6d** (a), derivatized (*S*)-**6d** synthetized with MBP-YfaU-W23V (b), and derivatized (*R*)-**6d** synthetized with KPHMT-I212A (c). Conditions: CHIRALCELL® ID 4.6 x 250 mm column, 5 µm, flow rate 0.7 mL min^{-1} at 20 °C and UV detection (254 nm). Isocratic elution hexane:*i*PrOH 75:25.

5.3 Compound 6e

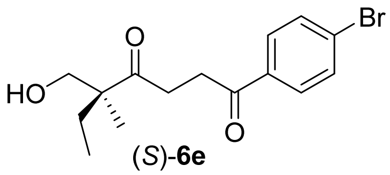

(*S*)-**6e**

a) *rac*-**6e**

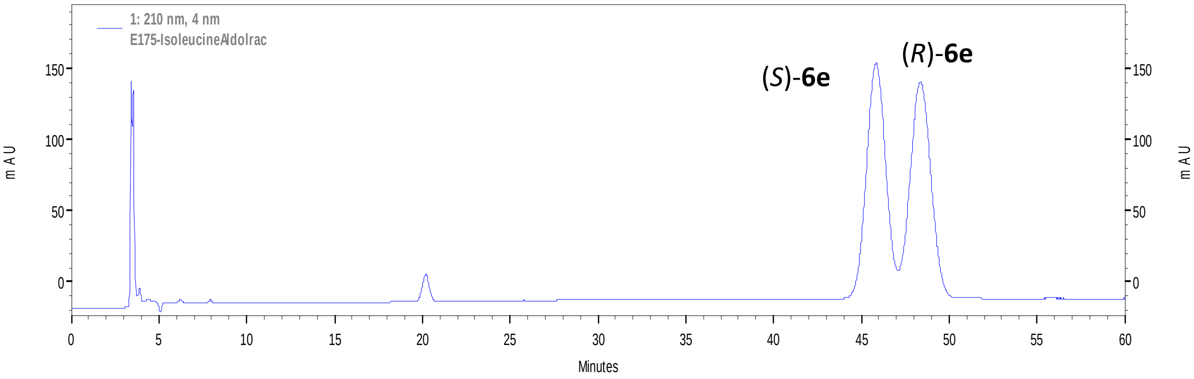

b) (*S*)-**6e**

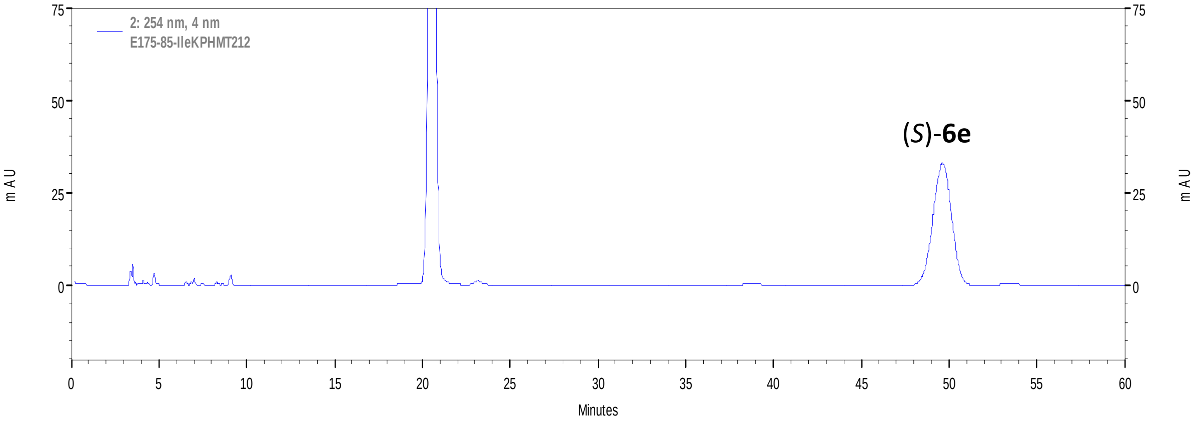

Figure S19. CSP-HPLC analysis chromatogram of *rac*-**6e** (a), and derivatized (*S*)-**6e** synthetized with KPHMT-I212A (b). Conditions: CHIRALPACK® ID 4.6 x 250 mm column, 5 µm, flow rate 0.7 mL min^{-1} at 20 °C and UV detection (254 nm). Isocratic elution hexane:iPrOH 75:25.

5.4 Compound 6g

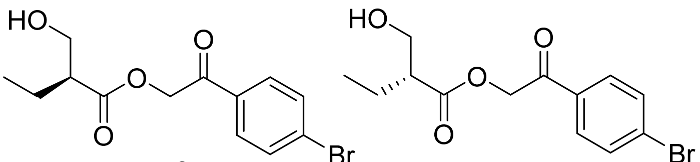

(*S*)-**6g** (*R*)-**6g**

a) *rac*-**6g**

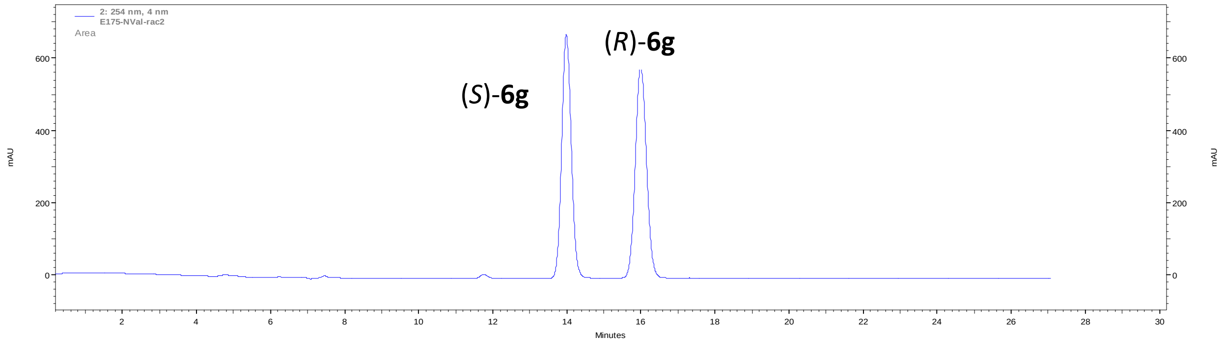

b) (*S*)-**6g**

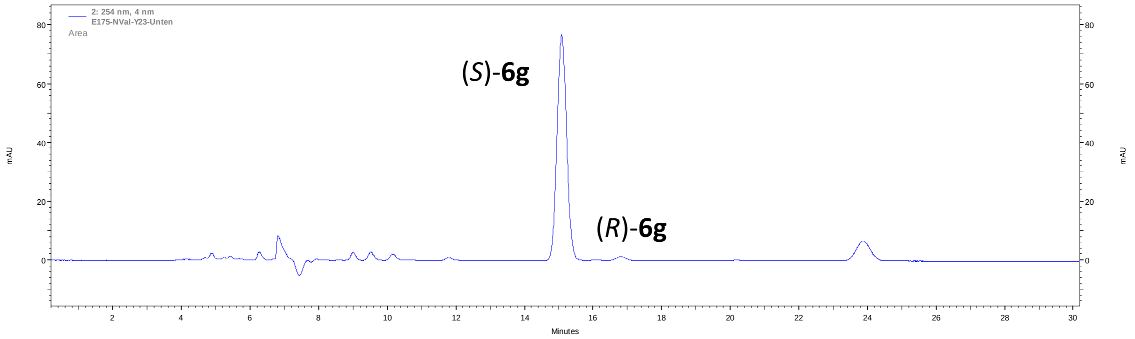

c) (*R*)-**6g**

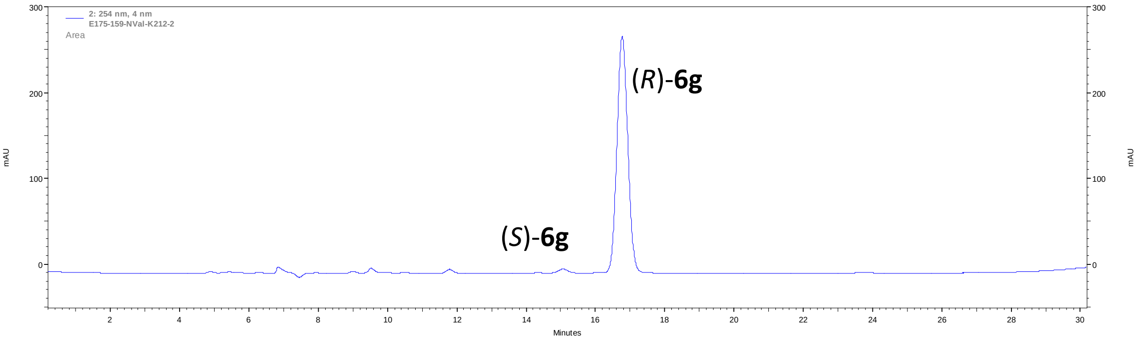

Figure S20. CSP-HPLC analysis chromatogram of *rac*-**6g** (a), derivatized (*S*)-**6g** synthetized with MBP-YfaU-W23V (b), and derivatized (*R*)-**6g** synthetized with KPHMT-*wt* (c). Conditions CHIRALPAK® IC 4.6 x 250 mm column, 5 μm, flow rate 0.7 mL min^{-1} at 20 °C and UV detection (254 nm). Isocratic elution hexane:iPrOH 75:25.

5.5 Compound 6h

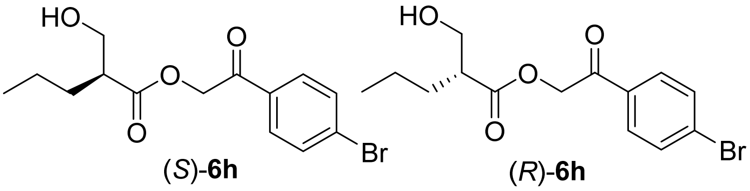

a) *rac*-**6h**

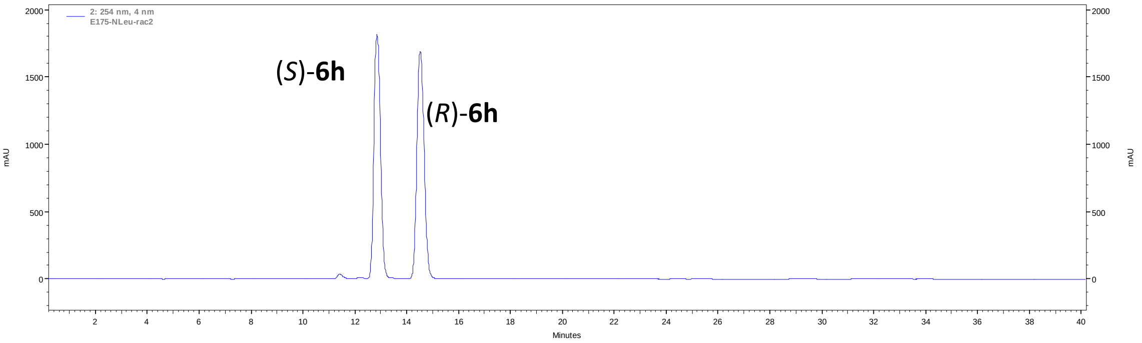

b) (*S*)-**6h**

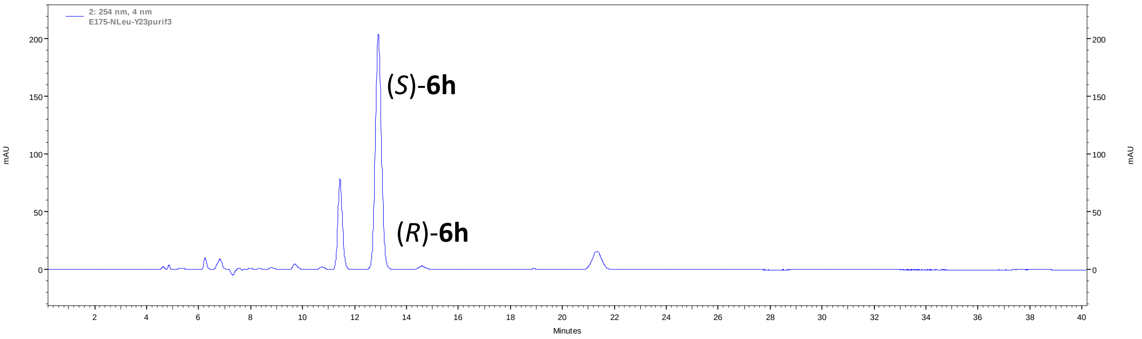

c) (*R*)-**6h**

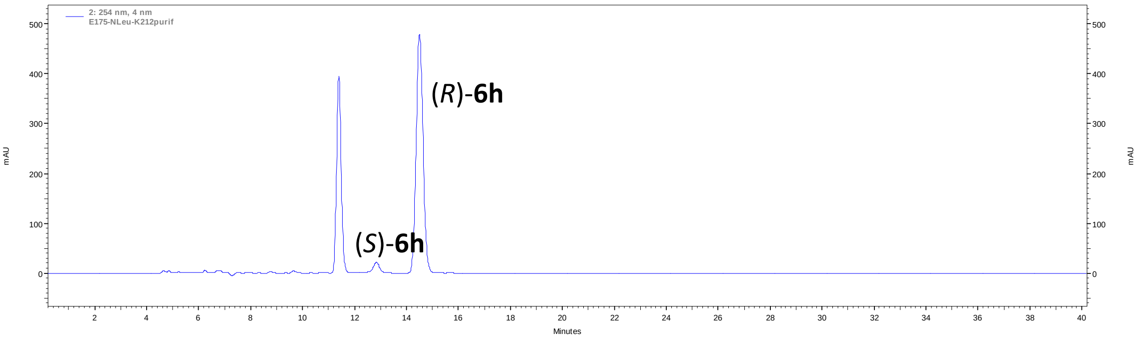

Figure S21. CSP-HPLC analysis chromatogram of *rac*-**6h** (a), derivatized (*S*)-**6h** synthetized with MBP-YfaU-W23V (b), and derivatized (*R*)-**6h** synthetized with KPHMT-I212A (c). Conditions CHIRALPAK® IC 46 x 250 mm column, 5 µm, flow rate 0.7 mL min^{-1} at 20 °C and UV detection (254 nm). Isocratic elution hexane:iPrOH 75:25.

5.6 Compound 6i

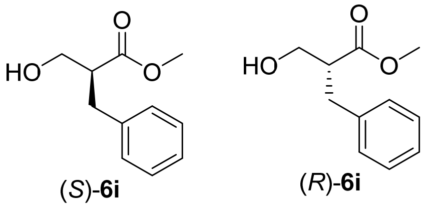

(*S*)-**6i** (*R*)-**6i**

a) *rac*-**6i**

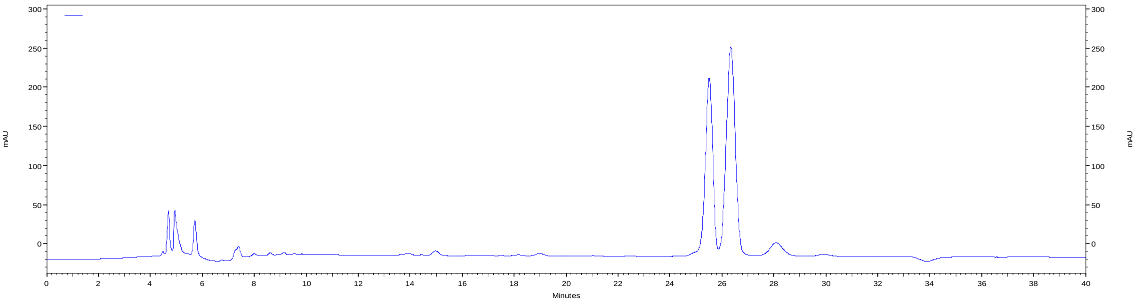

b) (*S*)-**6i**

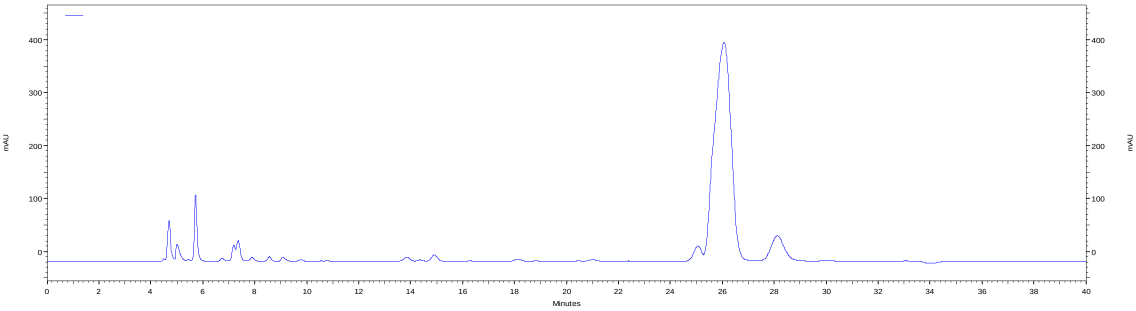

c) (*R*)-**6i**

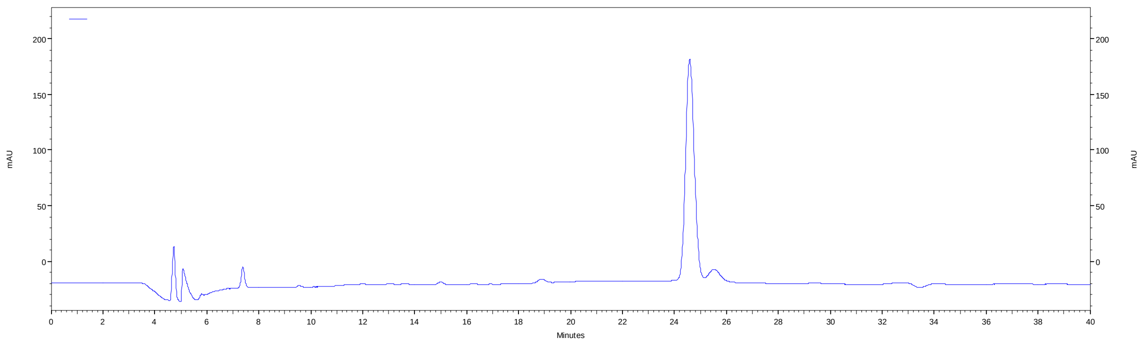

Figure S8. CSP-HPLC analysis chromatogram of *rac*-**6i** (a), derivatized (*S*)-**6i** synthetized with MBP-YfaU-W23V (b), and derivatized (*R*)-**6i** synthetized with KPHMT-I212A (c). Conditions CHIRALPAK® IC 46 x 250 mm column, 5 µm, flow rate 0.7 mL min^{-1} at 20 °C and UV detection (254 nm). Isocratic elution hexane:iPrOH 95:5.

6 NMR spectra.

a)

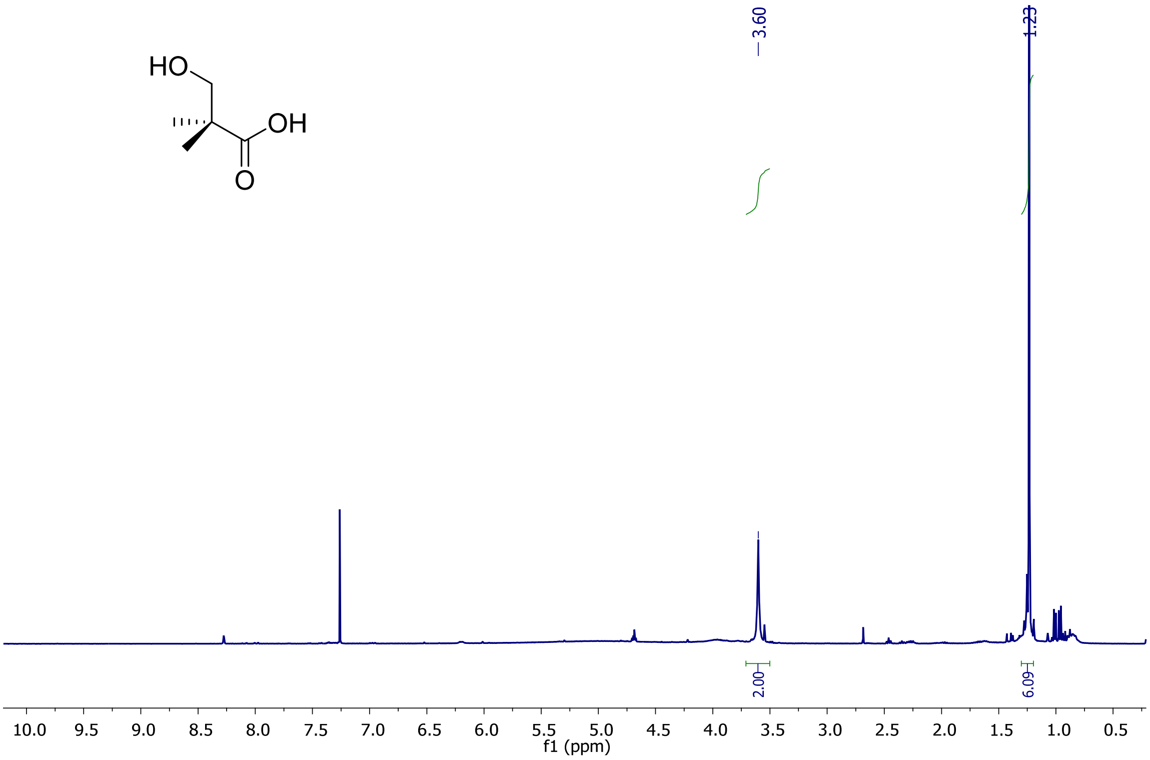

b)

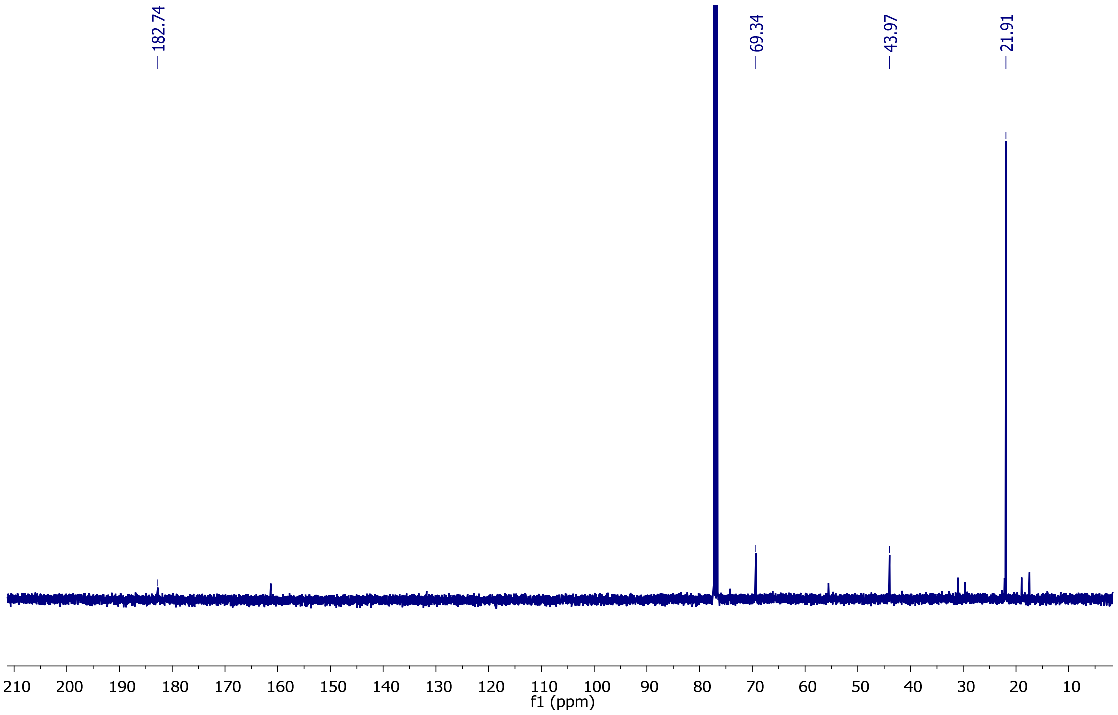

Figure S9. NMR (400 MHz) spectra ($CDCl_3$) of 3-hydroxy-2,2-dimethylpropanoic acid (**5a**): a) ^{1}H, and b) ^{13}C.

a)

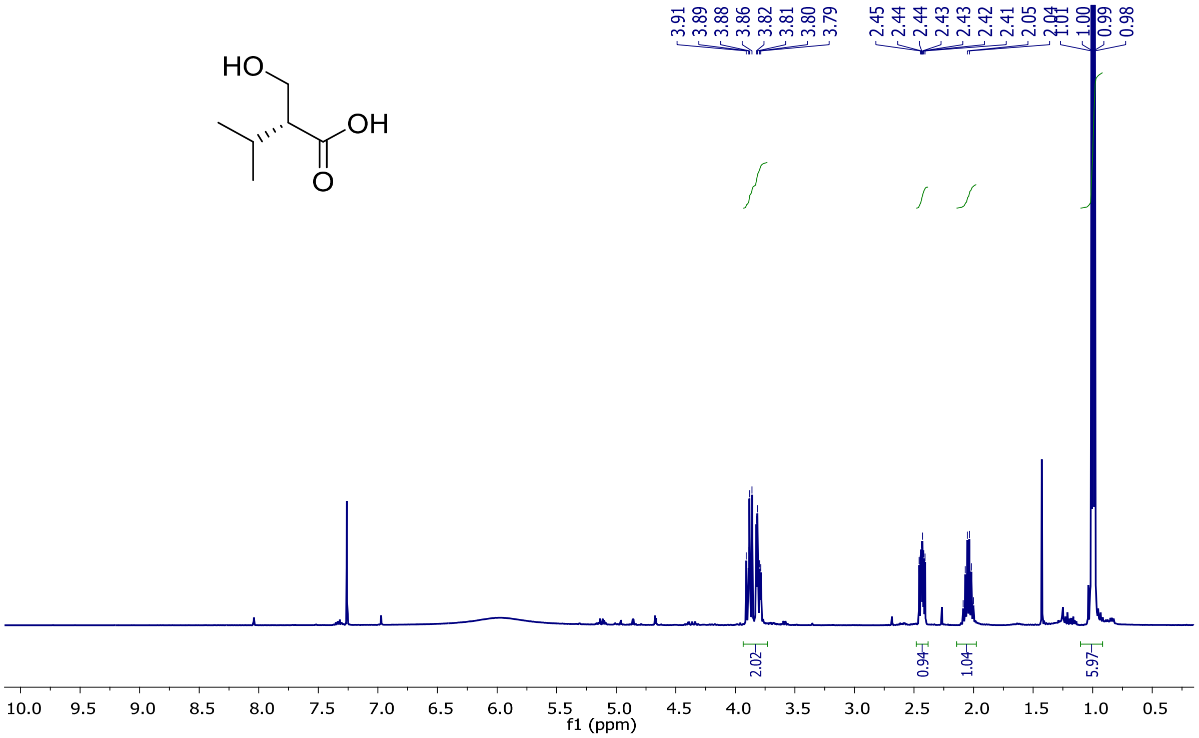

b)

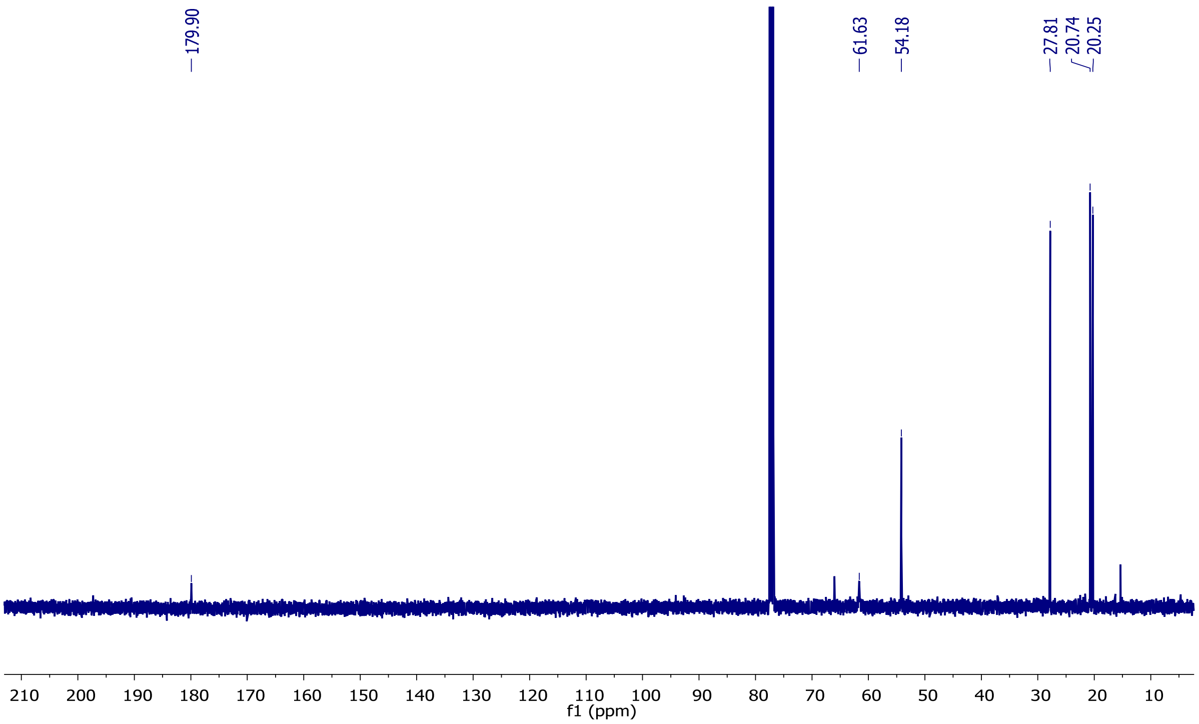

Figure S10. NMR (400 MHz) spectra ($CDCl_3$) of (*R*)-2-(hydroxymethyl)-3-methylbutanoic acid (**5c**): a) ^{1}H, and b) ^{13}C.

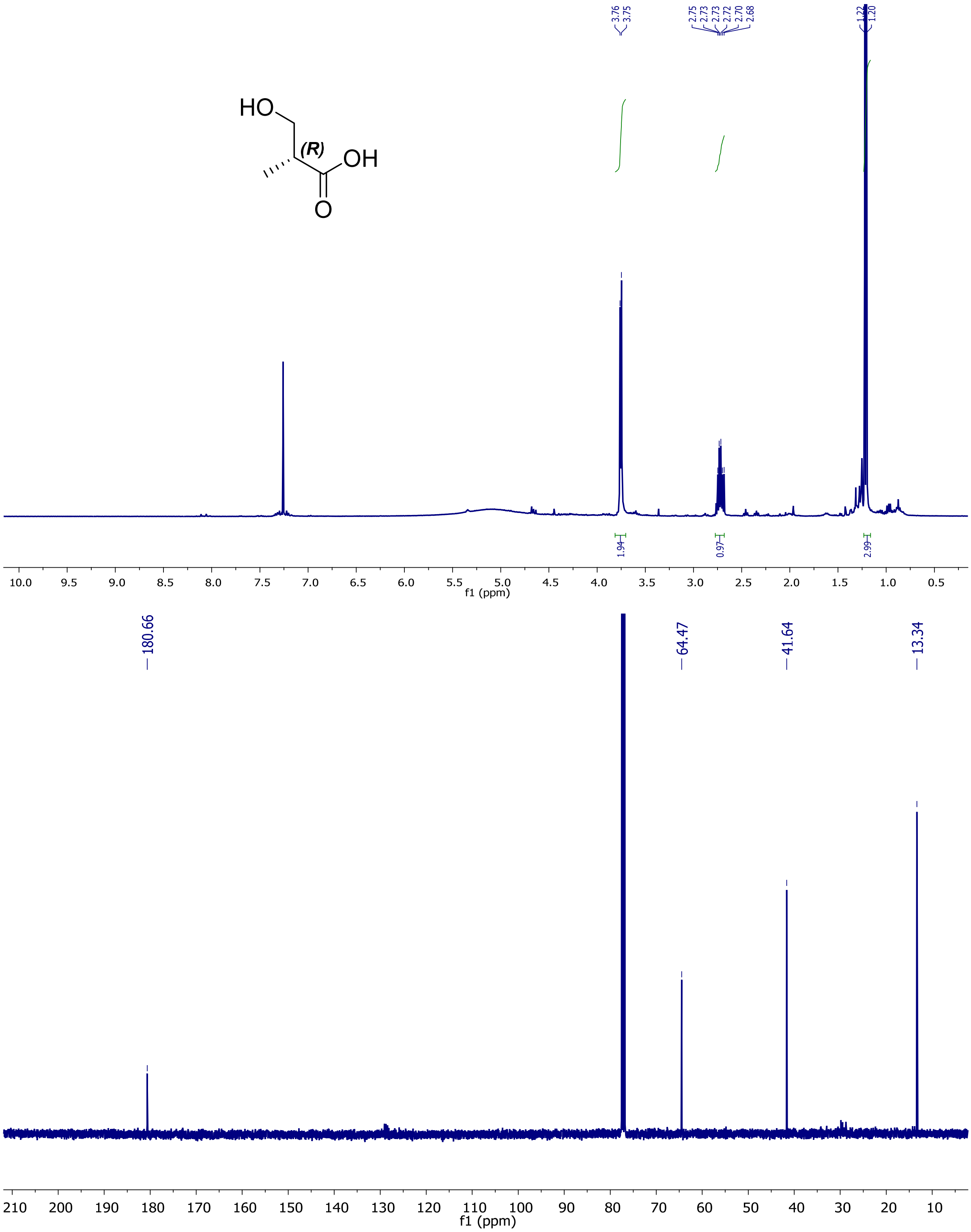

Figure S11. NMR (400 MHz) spectra ($CDCl_3$) of (*R*)-3-hydroxy-2-methylpropanoic acid (**5d**): a) ^{1}H, and b) ^{13}C.

a)

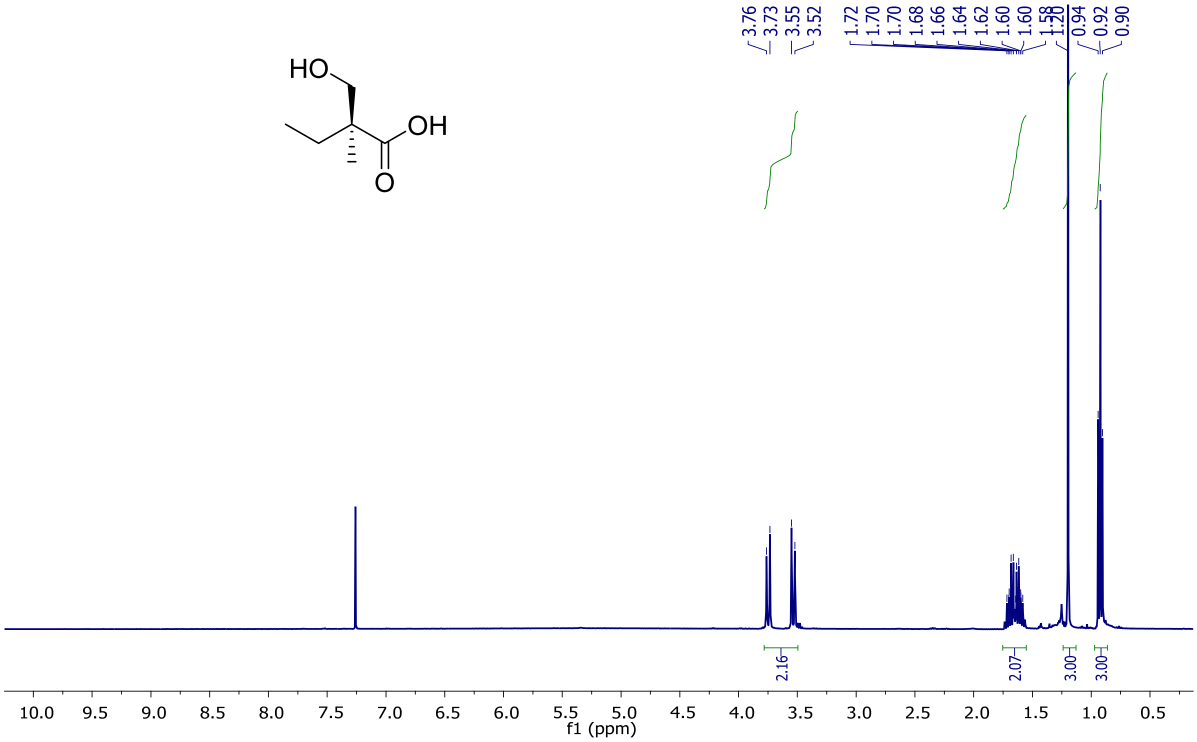

b)

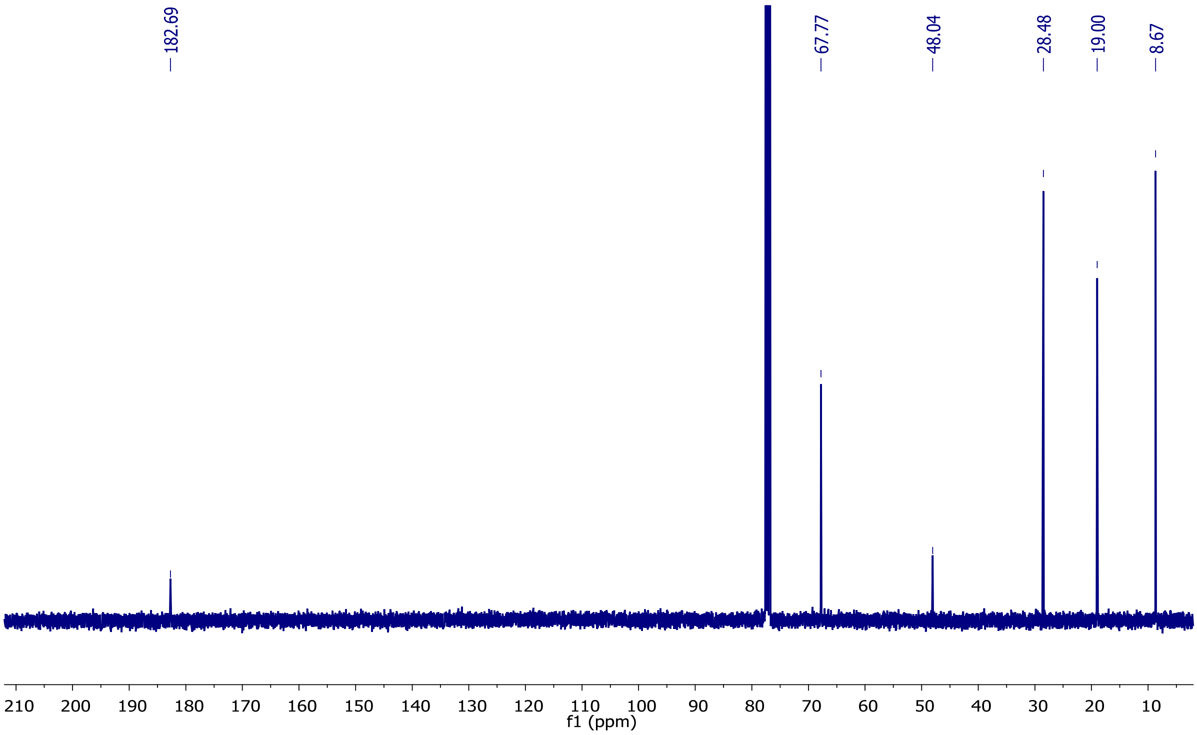

Figure S12. NMR (400 MHz) spectra ($CDCl_3$) of (*R*)-2-(hydroxymethyl)-2-methylbutanoic acid (**5e**): a) ^{1}H, and b) ^{13}C.

a)

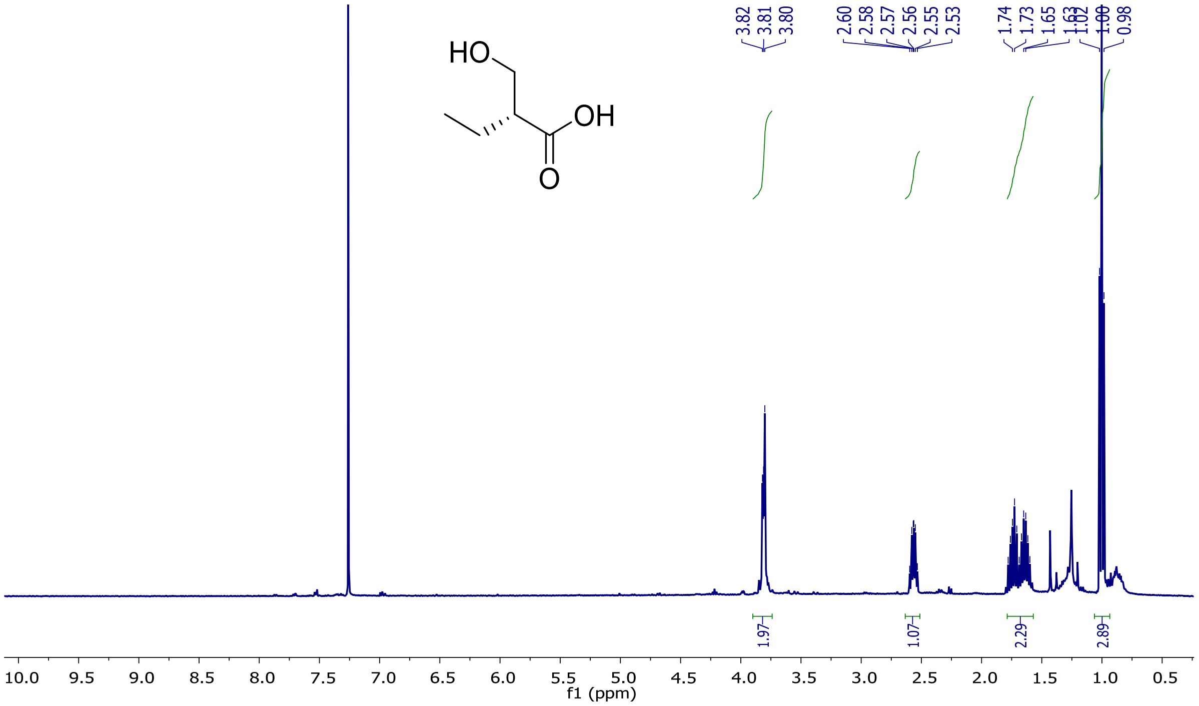

b)

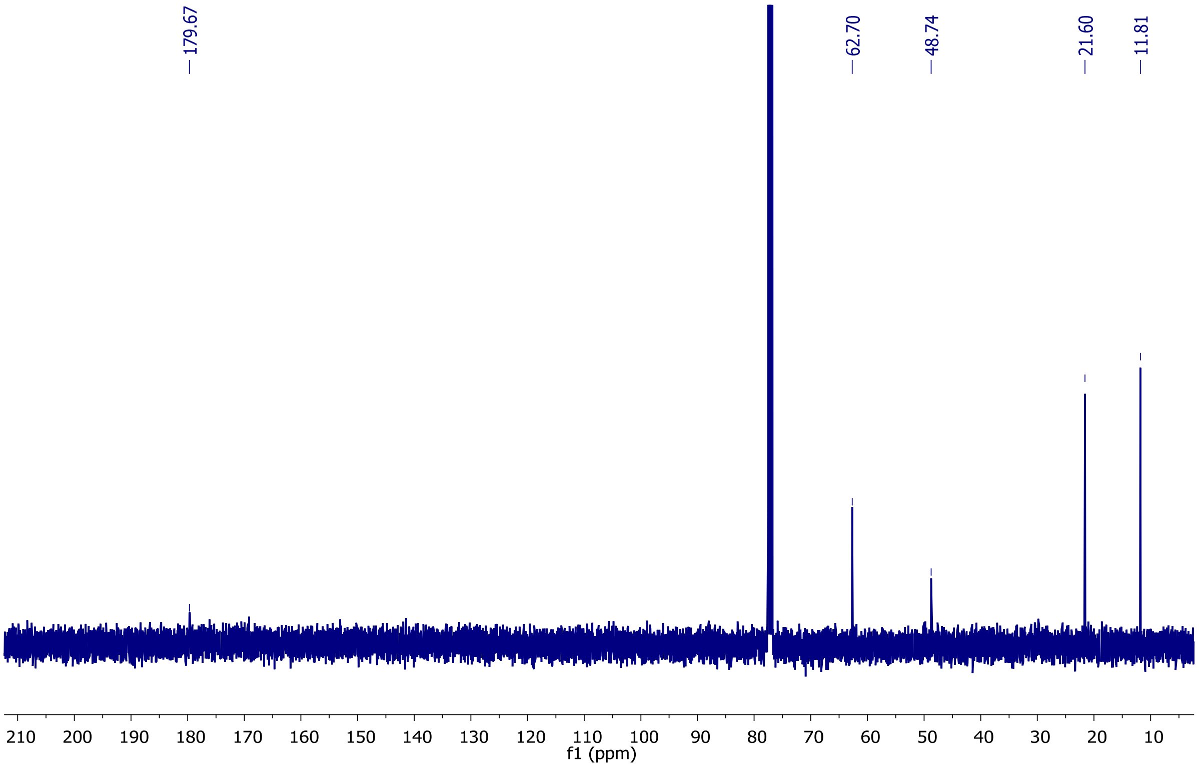

Figure S13. NMR (400 MHz) spectra ($CDCl_3$) of (*R*)-2-(hydroxymethyl)butanoic acid (**5g**): a) ^1H, and b) ^{13}C.

a)

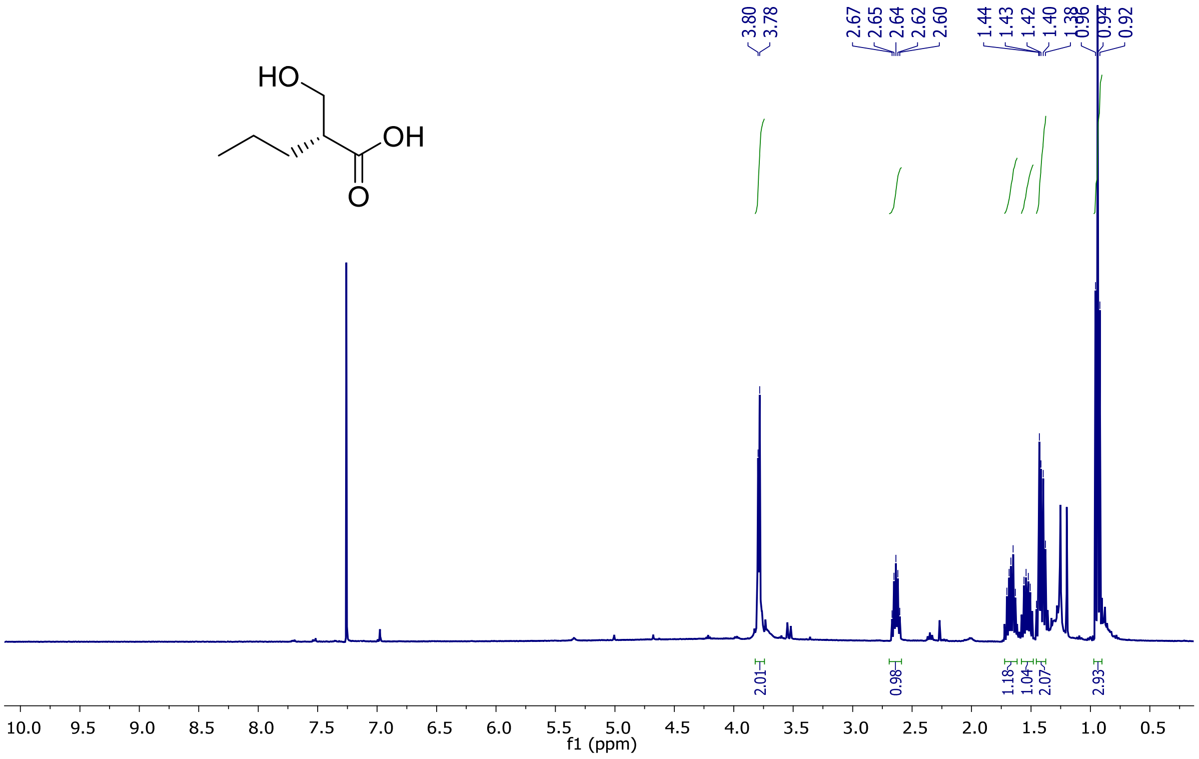

b)

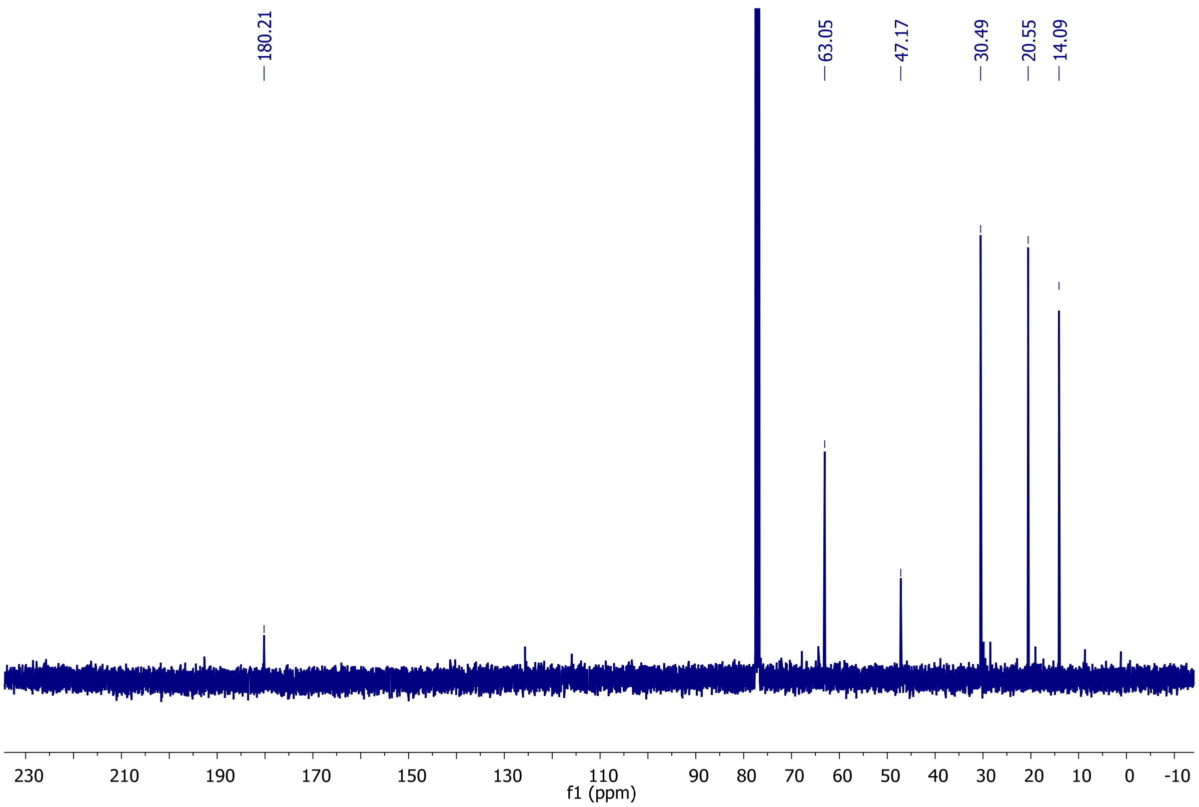

Figure S14. NMR (400 MHz) spectra ($CDCl_3$) of (*R*)-2-(hydroxymethyl)pentanoic acid (**5g**): a) ^1H, and b) ^{13}C.

a)

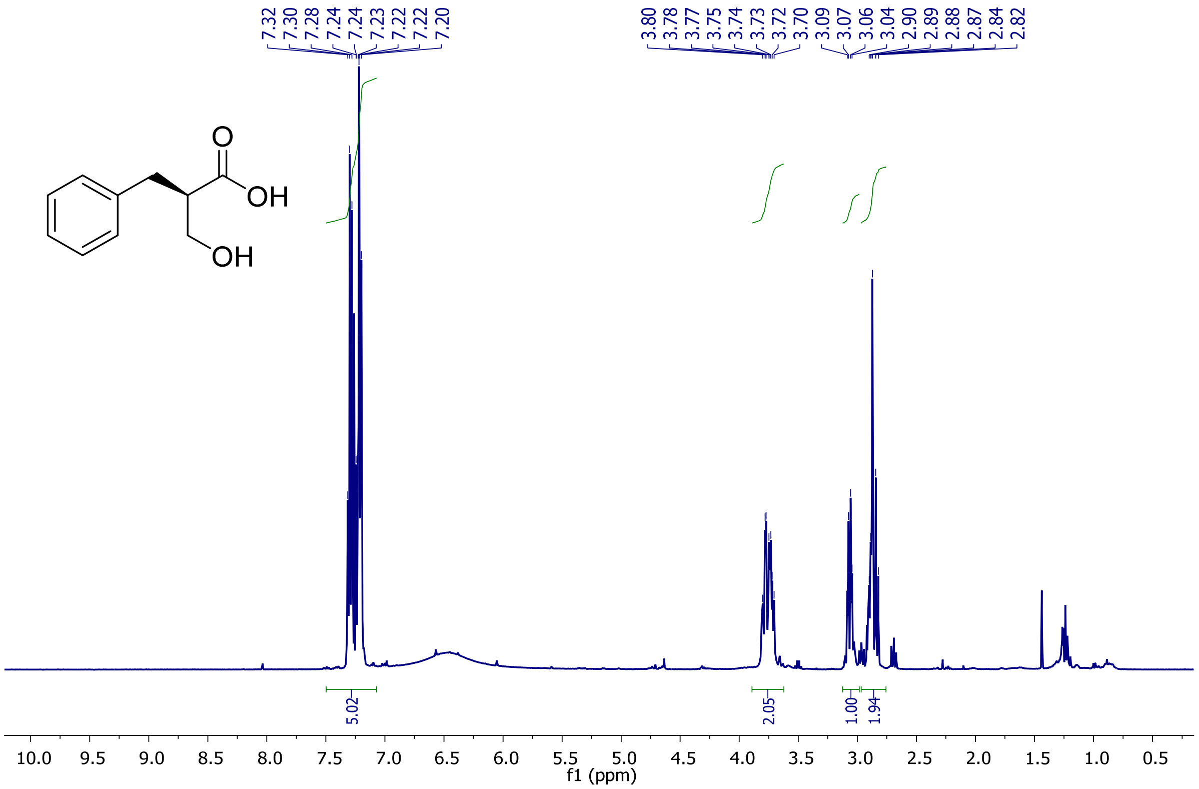

b)

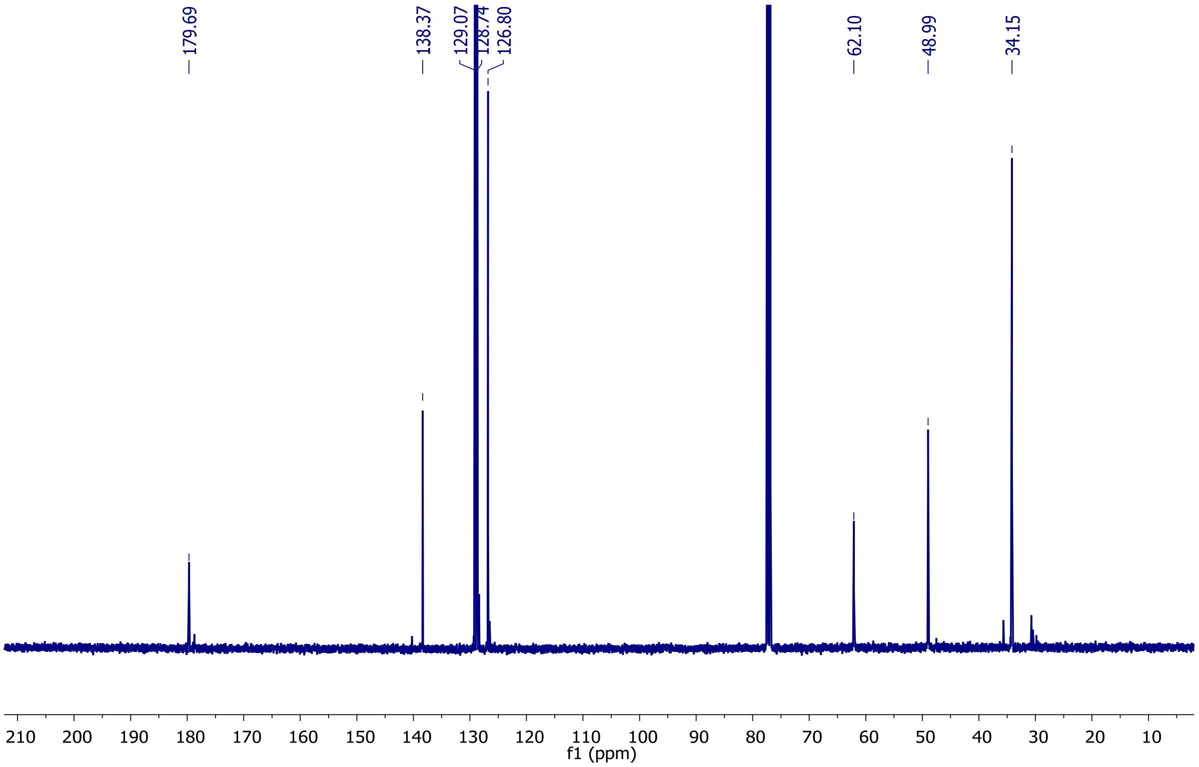

Figure S159. NMR (400 MHz) spectra ($CDCl_3$) of (*R*)-2-benzyl-3-hydroxypropanoic acid (**5i**): a) ^{1}H, and b) ^{13}C.
